# Acute gastrointestinal graft-versus-host disease is associated with reductions of secondary bile acids following allogeneic hematopoietic cell transplantation

**DOI:** 10.3389/fmicb.2026.1818647

**Published:** 2026-07-09

**Authors:** Robin Cagle, Sean Proll, Samuel S. Minot, Hayley Purcell, Wentao Zhu, Danijel Djukovic, Congzhou Liu, Tina Fiedler, Martha DeMeules, Marco Mielcarek, Sujatha Srinivasan, Daniel Raftery, Michael Wu, Steven A. Pergam, David N. Fredricks

**Affiliations:** 1 Vaccine and Infectious Disease Division, Fred Hutchinson Cancer Center, Seattle, WA, United States; 2 Department of Microbiology, University of Washington, Seattle, WA, United States; 3 Northwest Metabolomics Research Center, Department of Anesthesiology and Pain Medicine, University of Washington, Seattle, WA, United States; 4 Clinical Research Division, Fred Hutchinson Cancer Center, Seattle, WA, United States; 5 Division of Hematology and Oncology, University of Washington, Seattle, WA, United States; 6 Public Health Sciences Division, Fred Hutchinson Cancer Center, Seattle, WA, United States; 7 Biostatistics Program, Fred Hutchinson Cancer Center, Seattle, WA, United States

**Keywords:** *bai* operon, bile acid metabolism, bile salt hydrolase, graft-versus-host disease, gut microbiome, hematopoietic cell transplant, metabolomics, metagenomics

## Abstract

**Introduction:**

Allogeneic hematopoietic cell transplantation (HCT) can cure hematologic malignancies, but 30-70% of recipients experience acute graft-versus-host disease (GvHD). GvHD is associated with perturbations in the gut microbiome. Bile acids are host derived compounds that are transformed by gut bacteria and bind to specific host cell receptors, informing our hypothesis that changes in bile acid-metabolizing gut bacteria alter bile acid levels to affect gut physiology and immunity during GvHD.

**Methods:**

In a longitudinal case-control study of patients with and without acute gut GvHD, we characterized bile acid concentrations and the gut microbiome in stool.

**Results:**

Primary and conjugated bile acid levels were similar regardless of gut GvHD status, but endogenous secondary bile acid concentrations were associated with gut GvHD (*p* = 0.009). We observed 4.4-fold lower levels of endogenous secondary bile acids in GvHD, particularly lithocholic acid and derivatives (*p* = 0.004/p_adjusted_ = 0.02, fold change (FC) = 0.23). There was a 100-fold lower median abundance (*p* = 0.002 and FC < 0.01) and 20-fold lower median diversity of bacterial bile acid 7α-dehydroxylation (*bai*) genes (*p* = 0.0007 and FC < 0.05) in patients with GvHD.

**Discussion:**

This provides evidence that acute gut GvHD patients are deficient in microbial *bai* genes that make secondary bile acids.

## 1 Introduction

Allogeneic hematopoietic cell transplantation (HCT) is a potentially curative treatment for patients with hematologic malignancies and non-malignant hematologic disorders. However, significant morbidity and mortality are associated with acute graft-versus-host disease (GvHD) that affects 30–70% of HCT recipients. While the graft-versus-leukemia effect may help eradicate malignancy, detrimental GvHD occurs when allogeneic donor T cells attack healthy host epithelial tissues. Acute GvHD most commonly affects the skin, liver, or gut, though other tissues can be involved. Staging of acute gut GvHD is primarily based on the volume of diarrhea per day but is also characterized by increased apoptosis and intestinal crypt destruction on a gut biopsy. Overall GvHD grade is determined by the organs involved and stage of GvHD at each site (Glucksberg et al., 1974; Jagasia et al., 2015; Przepiorka et al., 1995).

Low gut microbial diversity has been linked to GvHD in numerous studies (Nalle and Turner, 2015; Nalle et al., 2019; Rafei and Jenq, 2020; Shono et al., 2015; Hill and Koyama, 2020; Holler et al., 2014; Malard et al., 2018). GvHD is marked by loss of intestinal barrier integrity and inflammation (Nalle and Turner, 2015; Noth et al., 2011; Johansson and Ekman, 2007; Hanash et al., 2012; Eriguchi et al., 2012; Weber et al., 2017). The gut microbiota and their metabolites may influence GvHD risk through impacts on the intestinal barrier and immune cell populations (Nalle and Turner, 2015; Noth et al., 2011; Johansson and Ekman, 2007; Hanash et al., 2012; Stoma et al., 2021; Weber et al., 2017). Recent studies have linked microbially-metabolized bile acids with intestinal health, including GvHD (Lindner et al., 2024). Bile acids function as lipid emulsifiers in the intestinal tract, but research has elucidated their role as signaling molecules in the intestine, binding host cell membrane receptors like G-protein-coupled receptor TGR5 and sphingosine-1-phosphate receptor 2 (S1PR2) and nuclear receptors such as farnesoid X receptor (FXR), pregnane X receptor (PXR), and vitamin D receptor (VDR) (Ridlon et al., 2016). Particular bile acids, including isolithocholic acid, isodeoxycholic acid, and 3-oxolithocholic acid, can suppress T cell differentiation into Th_17_ cells and promote T_reg_ differentiation (Paik et al., 2022; Hang et al., 2019; Campbell et al., 2020). Isolithocholic acid and 3-oxolithocholic acid are both produced by human gut microbial commensals, such as *Eggerthella lenta* and *Clostridium citroniae* (Paik et al., 2022). Furthermore, isolithocholic acid is produced by *Bacteroides fragilis* and *Mediterraneibacter gnavus* (Paik et al., 2022), whereas isodeoxycholic acid is produced by *Mediterraneibacter gnavus* and *Clostridium scindens* (Campbell et al., 2020).

Bile acids are enterohepatically circulated; they are produced in the liver from dietary and host-biosynthesized cholesterol before undergoing several host-mediated biotransformations. They are excreted in bile ducts and stored in the gall bladder for transport to the intestine as needed (e.g., after a meal) (Supplementary Figure 1). Bile acids enter the intestine as primary bile acids, such as cholic acid (CA) or chenodeoxycholic acid (CDCA), conjugated to taurine or glycine. These conjugated, primary bile acids encounter a range of gut microbes, nearly all of which possess a choloylglycine hydrolase enzyme, also known as bile salt hydrolase enzyme (encoded by *bsh* genes). Canonically, bile salt hydrolases deconjugate bile acids in one step, freeing amino acids and creating unconjugated primary bile acids. Recent findings identified a novel ability of bile salt hydrolases to re-conjugate taurine or glycine back onto bile acids (Guzior et al., 2024). Furthermore, bile salt hydrolases can re-conjugate bile acids to a diverse range of compounds, including all amino acids, polyamines, 5-aminovalerate, aminobutyrate, and more (Mohanty et al., 2024). The *bsh* gene is highly diverse; exact *bsh* gene sequences vary by strain, and evolutionary relationships do not correlate with *bsh* sequence identity (Song et al., 2019). Furthermore, the capacity for bile acid metabolism is not consistent within a bacterial genus or species.

A large portion of bile acids is actively reabsorbed across the small intestinal barrier as unconjugated, primary bile acids and then recycled back to the liver. The remaining bile acid pool migrates along the intestine, encountering more gut bacteria, including microbes like *Clostridium scindens* and others that possess the *bile acid inducible* (*bai*) gene operon that encodes a series of enzymes required for the multi-step conversion of unconjugated, primary bile acids into secondary bile acids by removing a hydroxyl group from the seventh carbon (7α-dehydroxylation) (Ridlon et al., 2016; Hylemon et al., 1991; Daniel and Ridlon, 2025). The majority of these unconjugated, secondary bile acids are passively reabsorbed across the intestinal barrier (95%) for recirculation back to the liver, whereas a smaller portion is excreted into stool (5%) (Pitt and Nakeeb, 2017; Devlin and Fischbach, 2015).

The human host and gut microbiota chemically modify bile acids, but transformations between primary vs. secondary and conjugated vs. unconjugated are key because these unique forms activate cell receptors differently (Ding et al., 2015; Wan and Sheng, 2018; Hanafi et al., 2018; Lee et al., 2022). Receptors include those on cell membranes (e.g., TGR5) and in the nucleus (e.g., FXR) in intestine-resident cells, like intestinal epithelial cells (IECs), or immune cells, such as T cells (Lindner et al., 2024; Paik et al., 2022; Hang et al., 2019; Campbell et al., 2020; Ding et al., 2015; Wan and Sheng, 2018; Hanafi et al., 2018). A previous study showed that T cell-induced inflammation alters microbial bile acid metabolism, contributing to bile acid dysbiosis that exacerbates GvHD (Lindner et al., 2024). This study investigates the relationships among bile acid disturbances, the gut microbiota, and GvHD in humans, considering how diverse bile acids may modulate GvHD or serve as markers of it. An enhanced understanding of gut microbiota dynamics and bile acid transformations/flux may pave the way to therapies that reduce GvHD and improve HCT outcomes.

## 2 Materials and methods

### 2.1 Study population and sample collection

The larger study population consisted of 329 patients undergoing allogeneic HCT at the Fred Hutchinson Cancer Center in Seattle, WA, between April 2013 and March 2020, who participated in a study examining GvHD and the gut microbiota with prospective collection of weekly stool samples under IRB#2608 with written informed consent. Sex as a biological variable was accounted for in this study by including a similar proportion of men and women participants in the case and control groups. Similar findings are reported for both sexes. Weekly kits that contained polyurethane foam swabs (Puritan Medical, Guilford, ME, USA) were provided to participants to self-collect stool samples at home; nurses collected swabs from inpatients. Swabs were returned to our laboratory by courier on the same day or by patients using insulated containers and ice packs if more than a day. Upon arrival, samples were tagged and stored at −80 °C until used for DNA extraction and metabolomics.

### 2.2 Experimental design

The goal of this study is to test the hypothesis that microbially metabolized bile acids contribute to gut GvHD initiation and progression, to understand better why some HCT recipients develop severe gut GvHD, whereas others do not. The study also identifies bile acid targets to investigate for potential therapeutic application to protect against gut GvHD. To accomplish this goal, we performed targeted bile acid metabolomics to measure bile acid concentrations, metagenomics to capture relative levels of microbial bile acid metabolic genes, and 16S rRNA sequencing to characterize bacterial communities in stool samples from HCT recipients, who did and did not develop GvHD post-HCT. Stool samples were collected approximately weekly, but this study’s sample sizes were informed by key time points in the HCT timeline (pre-HCT, 30 days post-HCT, and 60 days post-HCT) and the number of HCT recipients who developed GvHD and were suitably matched to an HCT recipient that did not develop GvHD along clinical and demographic criteria. The same cohort of participants and stool samples were independently analyzed, and results were integrated for comparison after each analysis was completed. No data from these analyses were excluded from this study.

### 2.3 Case–control design

From the greater study population of 329 allogeneic HCT recipients at the Fred Hutchinson Cancer Center, participants with stage 2–4 acute gut GvHD were selected and matched 1:1 to participants without gut GvHD (stage 0). Forty-nine participants with stage 2–4 acute gut GvHD were matched to a control participant without gut GvHD. Although GvHD is a time-dependent variable, we treated GvHD status as a binary variable for this study because there is no time-dependency in the no-GvHD group to match on. The median time to GvHD diagnosis within the GvHD case group was 25 days (Supplementary Figure 2). The criteria for matching/pairing participants included degree of human leukocyte antigen (HLA) matching, conditioning regimen, stem cell source, and GvHD prophylaxis/immunosuppression. Stool samples from the selected participants were collected at three time points, plus or minus 7 days; the pre-transplant time point reflects baseline, day 30 post-transplant represents the impact of transplant and the HCT preparative regimen, and day 60 post-transplant represents recovery from transplant.

### 2.4 DNA extraction

Bacterial DNA was extracted using the BiOstic Bacteremia DNA Isolation Kit (Qiagen, Germantown, MD, USA) as described previously (Srinivasan et al., 2024). Blank swabs were included as DNA extraction (negative) controls to monitor for contamination from extraction reagents.

### 2.5 Broad-range 16S rRNA gene PCR sequencing and taxonomic assignment

Bacterial DNA concentrations were measured as described previously (Srinivasan et al., 2012). Broad-range 16S rRNA gene PCR with Illumina sequencing targeting the V3-V4 region of the 16S rRNA gene was performed on all samples, as previously described (Srinivasan et al., 2024). A mock bacterial community from ATCC was also sequenced as a positive control to ensure the performance of laboratory methods and the bioinformatics pipeline. Negative controls for sequencing included both no-template PCR and DNA extraction controls to monitor for contamination. Raw sequence reads were processed using the DADA2 package (Callahan et al., 2016), and a list of unique sequence variants (SVs) was generated. Sequence reads are available from the NCBI Short Read Archive (Bioproject PRJNA1281039). Sequences were classified using a reference set tailored for the human gut microbiota (Golob et al., 2017). Taxonomy was assigned to each unique SV by phylogenetic placement (*pplacer*). Bacterial taxa represented by fewer than 25 reads in a sample were removed to minimize the inclusion of environmental contaminant sequences in the final dataset.

### 2.6 Whole-genome metagenomic sequencing

Genomic DNA was quantified using the Quant-iT™ dsDNA Assay Kit, high sensitivity (HS) (Thermofisher Scientific; Waltham, MA, USA). Sequencing libraries were prepared from 625 pg of genomic DNA and the quarter reaction workflow using the Illumina Nextera-XT library prep (Illumina; San Diego, CA, USA). The Illumina DNA/RNA UD indices and 12-cycle indexing were performed. Indexed libraries were pooled by volume, and cleanup was performed using 0.8X Agencourt AMPure XP beads (Beckman Coulter; Indianapolis, IN, USA). Size distribution within the library pool was validated using an Agilent High Sensitivity D5000 Screen Tape on the Agilent 4200 TapeStation (Agilent Technologies Inc., Santa Clara, CA, USA). Additional library QC and cluster optimization were done using an Invitrogen Qubit® 2.0 Fluorometer (Life Technologies; Carlsbad, CA, USA). Sequencing was performed on a NovaSeq 6000 instrument using an S2 flow cell with paired-end, 150-bp reads.

### 2.7 Metagenomic analysis

#### 2.7.1 Targeted metagenomic gene analysis

We curated a database of microbial bile acid metabolic genes of interest: *bile salt hydrolase (bsh)*, encoding cholylglycine hydrolase enzymes that can re-/deconjugate bile acids, and the *bile acid inducible (bai)* operon, encoding the enzymes needed to 7α-dehydroxylate bile acids. Two distinct approaches were adopted for the custom *bsh* and *bai* gene databases used as search criteria in the metagenomic data because *bsh* gene sequences are more diverse than *bai* gene sequences. *Bsh* gene database/catalog: Since *bsh* gene sequences are diverse, a sequence-based search would inherently limit the breadth of *bsh* genes included. Thus, we performed a keyword search for “choloylglycine hydrolase” in February 2025 on the NCBI Protein database.^1^ We applied criteria restricting the search space to ‘Bacteria’ and ‘RefSeq’ as the source database to promote inclusion of on-target, non-redundant, data-validated protein sequences. At the time of the search, this yielded 9,768 sequences, which were downloaded and used as the *bsh* gene database/catalog for metagenomic analyses.

#### 2.7.2 *Bai* gene database/catalog

The *bai* genes are more similar in sequence identity and more conserved within taxa. This operon is composed of at least 7 *bai* genes, which are well defined in *Clostridium scindens* (Funabashi et al., 2020). The *baiA2, baiB, baiCD, baiE, baiF, baiG*, and *baiH* genes from *C. scindens* were used as search criteria in April 2025 on the NCBI BLAST^2^ blastp program (Camacho et al., 2009; Altschul et al., 1990) and all sequences with >49% sequence identity were selected and included in the *bai* gene database/catalog. Using the microbial genes described above, the *gig-map* bioinformatics utility^3^ was used to generate a microbial gene catalog indexed for rapid alignment as follows:

1 Build Gene Catalog:

a Summary: Deduplicate the genes’ protein-coding sequences based on amino acid similarity clustering using CD-HIT.
b Nextflow workflow: FredHutch/gig-map/deduplicate.nf.
c Analysis parameters:

i cluster_coverage:0.9,
ii min_gene_length:50,
iii cluster_similarity:0.9

2 Align Reads to Genes:

a Summary: Quantify the number of reads that align to each gene in a precomputed catalog using the DIAMOND short read aligner.
b Nextflow workflow: FredHutch/gig-map/align_reads.nf
c Analysis parameters:

i max_evalue: 0.001
ii min_identity: 90
iii min_score_reads: 50

### 2.8 Targeted bile acid analysis by liquid chromatography-mass spectrometry (LC–MS)

The MS-based approach used here was adapted from previously reported methods (Kamp et al., 2021; Ginos et al., 2018). Human stool swab samples were thawed from −80 °C storage and thawed at 4 °C in 250 μL of cold MeOH/PBS (4:1, v/v), then vortexed for 60 s. A portion (200 μL) of the sample was transferred into a separate 2 mL Eppendorf vial, and 10 μL of isotope-labeled internal standard stock solution (composition of the internal standard stock solution can be found in Supplementary Table 1) was added, along with 600 μL methanol. These sample vials were vortexed again for 10 s and stored at −20 °C for 20 min. Vials were centrifuged at 18,000×*g* (at 4 °C) for 15 min. The supernatant (650 μL) was collected and placed in another 2 mL Eppendorf vial, then dried at 30 °C for approximately 2.5 h in a Speed Vac. Samples were reconstituted with 100 μL MeOH/water (1:1, v/v), vortexed for 10 s, then centrifuged for 5 min at 18,000×*g* (at 4 °C). The supernatants (80 μL) were transferred into LC vials for MS analysis.

A Waters Acquity I-Class UPLC TQS-micro MS (Waters, Milford, MA) was used for targeted MS analysis in negative ionization mode, and a Waters XSelect HSS T3 column kept at 40 °C was used for chromatographic separation. The mobile phase was composed of two solvents: 5 mM ammonium acetate in H_2_O with 0.1% acetic acid (A) and acetonitrile with 0.1% acetic acid (B). After 1 min of isocratic elution at 75% of solvent A, it was decreased to 5% at *t* = 15 min. Run solvent composition was then maintained at 5% for 10 min, followed by an increase to 75% at *t* = 25 min. The total experimental time for each injection was 40 min. A total of 55 bile acids were targeted, of which 32 had measurable signals and were analyzed. The five stable isotope (deuterium)-labeled internal standards were used to determine the bile acid molar amounts (Raftery, 2014; Sarafian et al., 2015).

### 2.9 Bile acid dataset from a similar center

Publicly available data were downloaded from the Lindner et al. research article published in 2024 (Lindner et al., 2024). Metadata from sheet, ‘cohort_BAS,’ columns A–D was used with bile acid concentration data from sheet, ‘filtered_combined_table,’ column AK, ‘lithocholic_acid,’ and column AL, ‘lithocholic_acid_3_sulfate.’ The Mann–Whitney U test was used to compare the distribution of each bile acid’s concentration in GvHD cases vs. controls at peri-engraftment time points and post-engraftment for each participant.

### 2.10 Steroid (prednisone/methylprednisolone) analysis

Active prednisone/methylprednisolone therapy was defined as receiving any dose of prednisone or methylprednisolone on any day in the 7 days before the sample collection date. Samples that were collected while the participant was receiving steroid treatment were excluded in a separate sensitivity analysis to determine if prednisone may bias the measured bile acid levels.

### 2.11 Statistical analysis and software

**Figure 1:**
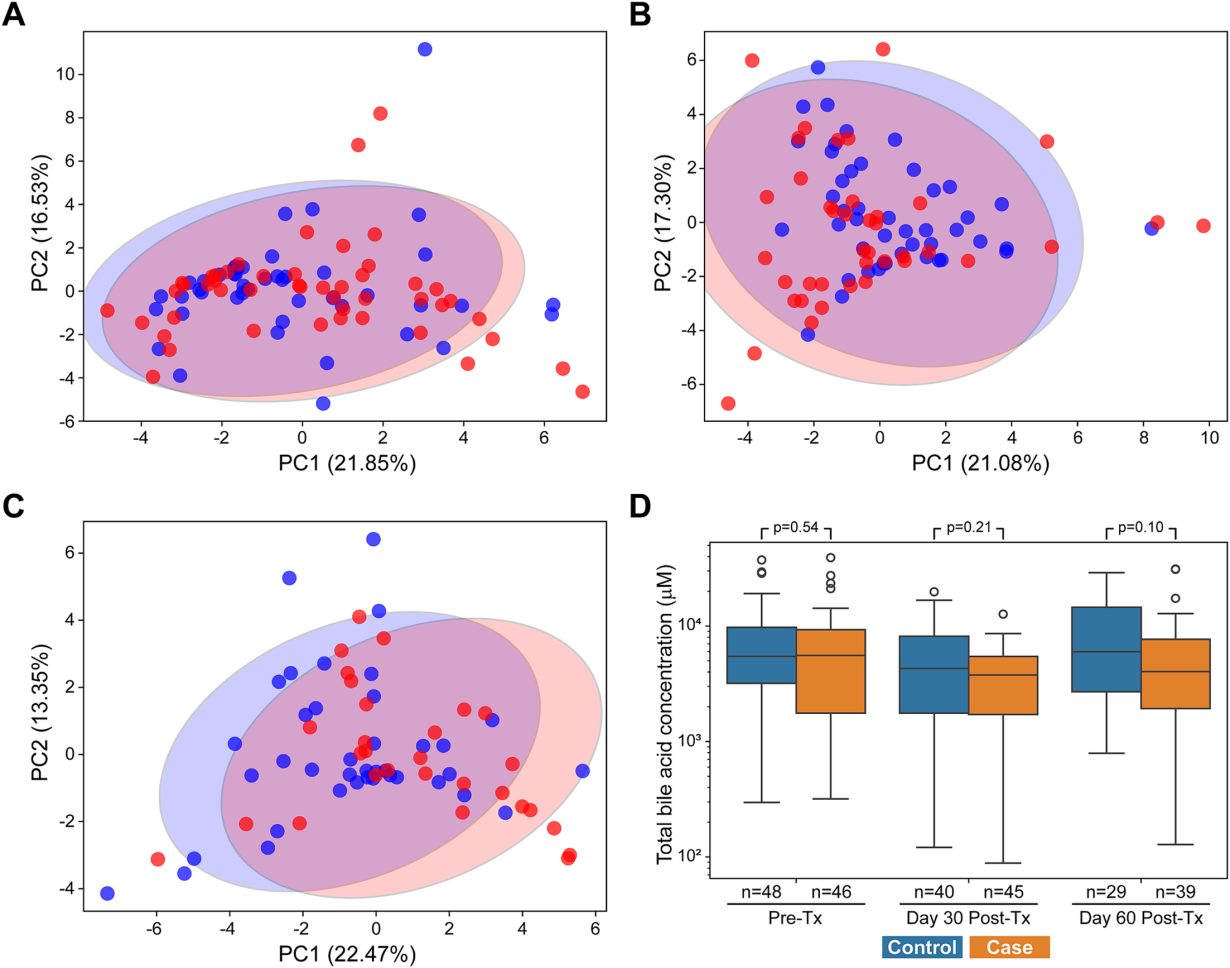
Global differences in BAs. Post-HCT, significant differences in global bile acid levels emerge. There were no global differences in BAs (reflecting the population of 32 measured BAs) between GvHD cases and controls as observed by principal component analysis (PCA) **(A)** pre-transplant. PCA visualized the statistically significant differences between GvHD cases’ and controls’ bile acid concentrations at **(B)** 30 days post-transplant (*p* = 0.008, *R*^2^ = 0.042) and **(C)** 60 days post-transplant (*p* = 0.05, *R*^2^ = 0.041). Values next to the component on each axis display the amount of variation attributed to that component. Cases are in blue, controls in red. **(D)** There were lower levels of summed BAs in cases at 60 days, although this was not statistically significant. The box represents the interquartile range (IQR): the lower bound represents the first quartile, the center bound represents the median, and the upper bound represents the third quartile. The lowest whisker represents the minimum and the highest whisker the maximum value, where observations outside of 1.5 * IQR are considered outlying values and displayed independently. PERMANOVA test was performed to generate *R*^2^ and *p*-values. PC = principal component. Tx = hematopoietic cell transplant.

The concentrations of bile acids, the sums or diversity of bile acid metabolism genes, and the abundances of microbial taxa, determined by 16S rRNA gene sequencing in stool samples from GvHD cases and no-GvHD samples at each time point, were analyzed for statistical significance using the Mann–Whitney U test since the means are non-parametric and non-evenly distributed. A 10% prevalence filter was applied to all samples after the microbial taxa abundance data were analyzed. Odds ratios and prevalence comparisons were analyzed for statistical significance using Fisher’s Exact test, as the prevalence counts are nonparametric. The strength of the correlational relationships was evaluated using Kendall–Tau correlation (more conservative vs. Spearman), given that the sample populations were non-parametric and non-evenly distributed. The data could be sparse with small values. A 10% prevalence filter was applied to the microbial taxa abundance and to the bile acid concentration data before calculating correlations. Where *p*-value adjustment for multiple comparisons was appropriate, the Benjamini-Hochberg false discovery rate (FDR) adjustment was used. Adjusted *p*-values ≥ 0.05 were considered significant. The Python pandas (version 2.2.3), numpy (2.1.1), and openpyxl (3.1.5) packages were used for data organization (McKinney, 2010; NumPy, 2024; Gazoni and Clark, n.d.). The Python statsmodels (0.14.4), skbio (version 0.7.2), sklearn (1.5.2), and scipy (1.14.1) packages were used for all statistical analyses (Perktold et al., 2024; SciPy, n.d.; Pedregosa et al., 2011; Aton et al., 2025). The following formula was used to calculate the *R*^2^ values in Figures 1A–C: *R*^2^ = 1/(1 + ((n − g)/((g − 1)*F))), where n = number of samples, g = number of groups, and F = the PERMANOVA F-prime statistic (Aton et al., 2025). Figures were created using matplotlib (3.9.2) and seaborn (0.13.2) (Waskom, 2021; Hunter, 2007). Comparisons of relative abundances of microbial taxa were performed in R using the Physloseq (1.50.0), microViz (0.12.6), ggplot2 (3.5.1), plyr (1.8.9), dplyr (1.1.4), RColorBrewer (1.1–3), and ggiraph (0.8.12) packages (McMurdie and Holmes, 2013; Barnett et al., 2021; Gohel and Skintzos, 2026; Wickham, 2011; Neuwirth, 2022; Wickham et al., 2026; Wickham, 2016).

### 2.12 Study approval

The study was Institutional Review Board (IRB) approved, and all patients provided written informed consent before participation.

### 2.13 Data availability

Original datasets are available in a publicly accessible repository: The original contributions presented in the study are publicly available. The 16S rRNA gene sequencing read data can be found in the NCBI Short Read Archive: Bioproject PRJNA1281039. The metagenomic sequencing read data will be made available/accessible in the NCBI Short Read Archive. The metabolomics data will be made available/accessible on the National Metabolomics Data Repository (NMDR)/Metabolomics Workbench. The supporting analytic code will be made available/accessible on GitHub.

## 3 Results

### 3.1 Study population

Demographic and clinical characteristics of the cases and controls were well matched (by design) except for the presence of acute gut GvHD (Table 1). Further details on the distribution of overall grade and individual organ stages of GvHD in case and control participants can be found in Supplementary Table 2.

**Table 1:** Demographics of the study population.

Characteristic	Characteristic categories	Cases (*n* = 49), *n* (%)	Controls (*n* = 49), *n* (%)
Age	Median	56 (20–75)	55 (26–72)
Race	Asian	2 (4.08)	2 (4.08)
	Black or African American	3 (6.12)	0 (0)
	White	41 (83.67)	43 (87.76)
	More than one race	1 (2.04)	3 (6.12)
	Unknown or not reported	2 (4.08)	1 (2.04)
Ethnicity	Hispanic or Latino	2 (4.08)	0 (0)
	Not Hispanic or Latino	46 (93.88)	48 (97.96)
	Unknown	1 (2.04)	1 (2.04)
Sex	Women	20 (40.82)	14 (28.57)
	Men	29 (59.18)	35 (71.43)
Donor relationship	Related	12 (24.49)	10 (20.41)
	Not related	37 (75.51)	39 (79.59)
HLA	Haploidentical	5 (10.2)	3 (6.12)
	Matched	36 (73.47)	37 (75.51)
	Cord	6 (12.24)	5 (10.2)
	Mismatch	2 (4.08)	4 (8.16)
Underlying diagnosis	Leukemia	27 (55.1)	27 (55.1)
	Myelofibrosis	4 (8.16)	5 (10.2)
	Aplastic anemia	9 (18.37)	8 (16.327)
	Lymphoma	4 (8.16)	6 (12.24)
	Other	5 (10.2)	3 (6.12)
Stem cell source	Bone marrow	3 (6.12)	3 (6.12)
	Cord	6 (12.24)	5 (10.2)
	Peripheral blood stem cell	40 (81.63)	41 (83.67)
Conditioning regimen	Myeloablative	23 (46.94)	21 (42.86)
	Reduced intensity	25 (51.02)	22 (44.90)
	Non-myeloablative	1 (2.04)	6 (12.24)
GvHD prophylaxis	CNI + methotrexate	13 (26.53)	17 (34.69)
	CNI + MMF	11 (22.45)	13 (26.53)
	CNI + MMF + rapamycin	8 (16.33)	4 (8.16)
	Other	17 (34.69)	15 (30.61)

The demographic and clinical characteristics of the cases and controls are well matched except for the presence of acute gut GvHD. The numbers in parentheses after the median age are the age range of participants of this study. CNI = Calcineurin-inhibitor, MMF = mycophenolate mofetil.

### 3.2 Global bile acid concentrations post-HCT

To examine global differences in bile acid concentrations between GvHD cases and no-GvHD controls, we first used principal component analyses (PCAs) based on the concentrations of each of the 32 bile acids measured in this study. We observed statistically significant differences between GvHD case and control samples on days 30 and 60 post-transplant (*p* = 0.008 and 0.05, *R*^2^ = 0.042 and 0.041, respectively; Figures 1B,C). Nevertheless, we observed overlap in covariance ellipses (Figures 1A–C), with similar median case and control concentrations of summed bile acids at each time point (Figure 1D). Examination of the change in bile acid levels over time in GvHD cases revealed that summed bile acid levels decreased 1.5-fold from 5.6 mM pre-transplant to 3.8 mM (*p* = 0.02, FC = 0.68) at day 30 post-transplant, whereas levels remained similar in no-GvHD controls (5.5 mM vs. 4.3 mM, *p* = 0.09; Figure 1D; Supplementary Figure 3A). We next sought to determine which bile acid groups contribute to the variability seen in the PCA plots.

### 3.3 Lower concentrations of endogenous secondary bile acids associate with GvHD post-HCT

**Figure 2:**
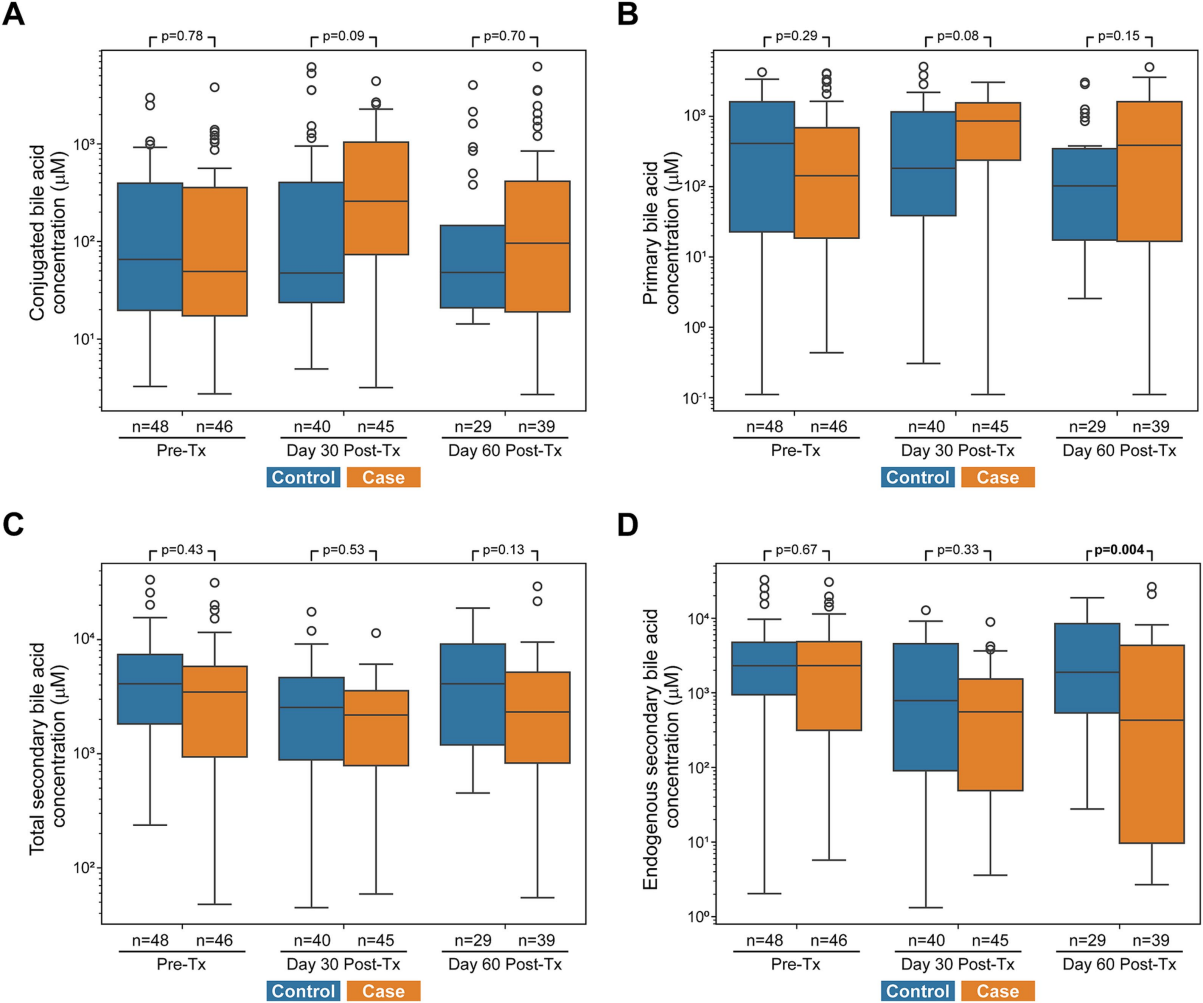
Group-level differences in BAs. Endogenous secondary bile acids are present at lower levels in GvHD cases vs. controls 60 days post-transplant. **(A)** There are no significant differences in conjugated BAs (12) between GvHD cases vs. controls at the 3 assessed time points. **(B)** There are no significant differences in primary BAs (6) between GvHD cases vs. controls. **(C)** There are no significant differences in secondary BAs (14) between GvHD cases vs. controls. **(D)** There are lower levels of endogenous secondary BAs (11) in GvHD cases vs. controls 60 days post-transplant. The number shown on each box represents the sample group’s median. The Mann–Whitney U test was performed to generate *p*-values. Raw/unadjusted *p*-values are shown. The FDR-adjusted *p*-value for panel D day 60 post-HCT is 0.02. The bounds of the boxes, the lines within the boxes, the whiskers, and the consideration of outlying values are the same as those defined in figure legend. Tx = hematopoietic cell transplant.

We examined the summed concentrations of three key groups of bile acids: conjugated, primary, and secondary bile acids (Supplementary Figure 1) that are influenced by bacterial metabolism. At first, it appeared that there were no significant differences in bile acid concentrations between GvHD cases vs. controls at any time point for the 12 conjugated, six primary, and 14 secondary bile acids measured (Figures 2A–C). However, levels of both conjugated and primary bile acids significantly increased in GvHD case samples from pre-transplant to day 30 post-transplant (*p* = 0.02, FC = 5.2 and 6.0, respectively; Supplementary Figures 3B,C), whereas secondary bile acid concentrations decreased 1.6-fold from pre-transplant to day 30 post-transplant in GvHD cases and no-GvHD controls (*p* = 0.05 and 0.05; FC = 0.63 and 0.62, respectively; Supplementary Figure 3D). Additionally, grouping all secondary bile acids ignores a key distinguishing factor in HCT recipients that we note below.

The exogenous secondary bile acid ursodeoxycholic acid (UDCA, also known as ursodiol) is administered to all allogeneic HCT recipients, regardless of GvHD status, to mitigate post-transplant liver complications, such as sinusoidal obstruction syndrome (SOS), also known as hepatic veno-occlusive disease (VOD). Ursodiol is administered from before conditioning through 90 days post-HCT. Although the intent behind UDCA clinical administration is to prevent liver damage, protection against acute gut GvHD has also been observed (Ruutu et al., 2002; Ruutu et al., 2014). Since all recipients in the study received exogenous UDCA, we excluded UDCA from the endogenous secondary bile acid group. When excluding UDCA and metabolites tauro-UDCA and glyco-UDCA, statistically significant differences in the 11 endogenous secondary bile acids were observed on days 30 and 60 post-HCT by PCA (*p* = 0.009 and 0.05, respectively; Supplementary Figures 4B,C). There was a 4.4-fold decrease in levels of endogenous secondary bile acids in GvHD case samples (median 430 μM) vs. no-GvHD samples (median 1,900 μM) on day 60 post-transplant (*p* = 0.004, p_adjusted_ = 0.02, FC = 0.23; Figure 2D; Supplementary Table 3). Endogenous secondary bile acid levels also significantly change over time within cases or controls. GvHD case median endogenous secondary bile acid levels were 2,300 μM pre-transplant and decreased 4.2-fold to 550 μM by 30 days post-transplant (*p* = 0.0007, FC = 0.24; Supplementary Figure 3E). Control (no-GvHD) median endogenous secondary bile acid levels were 2,300 μM pre-transplant and also decreased, but only 2.9-fold to 790 μM by 30 days post-transplant (*p* = 0.02, FC = 0.34; Supplementary Figure 3E). Although these endogenous secondary bile acid levels decreased in both cases and controls from pre-transplant to 30 days post-HCT, only the control endogenous secondary bile acid levels recovered by day 60 post-transplant to a median of 1,900 μM (*p* = 0.01, FC = 2.4; Supplementary Figure 3E). This contrasted with GvHD case endogenous secondary bile acid levels on day 60 post-HCT, median 430 μM, which remained 5.3-times lower than pre-transplant levels (*p* = 0.01, FC = 0.19). These data provided evidence of persistent deficiency of endogenous secondary bile acids during GvHD post-HCT (Supplementary Figure 3E).

We gained even greater insight by focusing on stool samples from 45 GvHD case participants on day 30 post-HCT. These samples were divided into two groups: those diagnosed with GvHD by the time of the day 30 sample collection time (*n* = 30, current GvHD) and those not yet diagnosed with GvHD at that time (*n* = 15, pre-GvHD). As a group, the summed levels of endogenous secondary bile acids on day 30 in the pre-GvHD cases were statistically indistinguishable from the no-GvHD group (*p* = 0.64; Supplementary Figure 6), whereas the current GvHD cases had 4-fold lower levels of endogenous secondary bile acids compared to the pre-GvHD group (*p* = 0.03/p_adj_ = 0.22, FC = 0.25; Supplementary Figure 6; Supplementary Table 4). These data provide further evidence that endogenous secondary bile acid levels are linked to GvHD status.

### 3.4 Low concentrations of lithocholic acid and similarly structured secondary bile acids associate with GvHD

**Figure 3:**
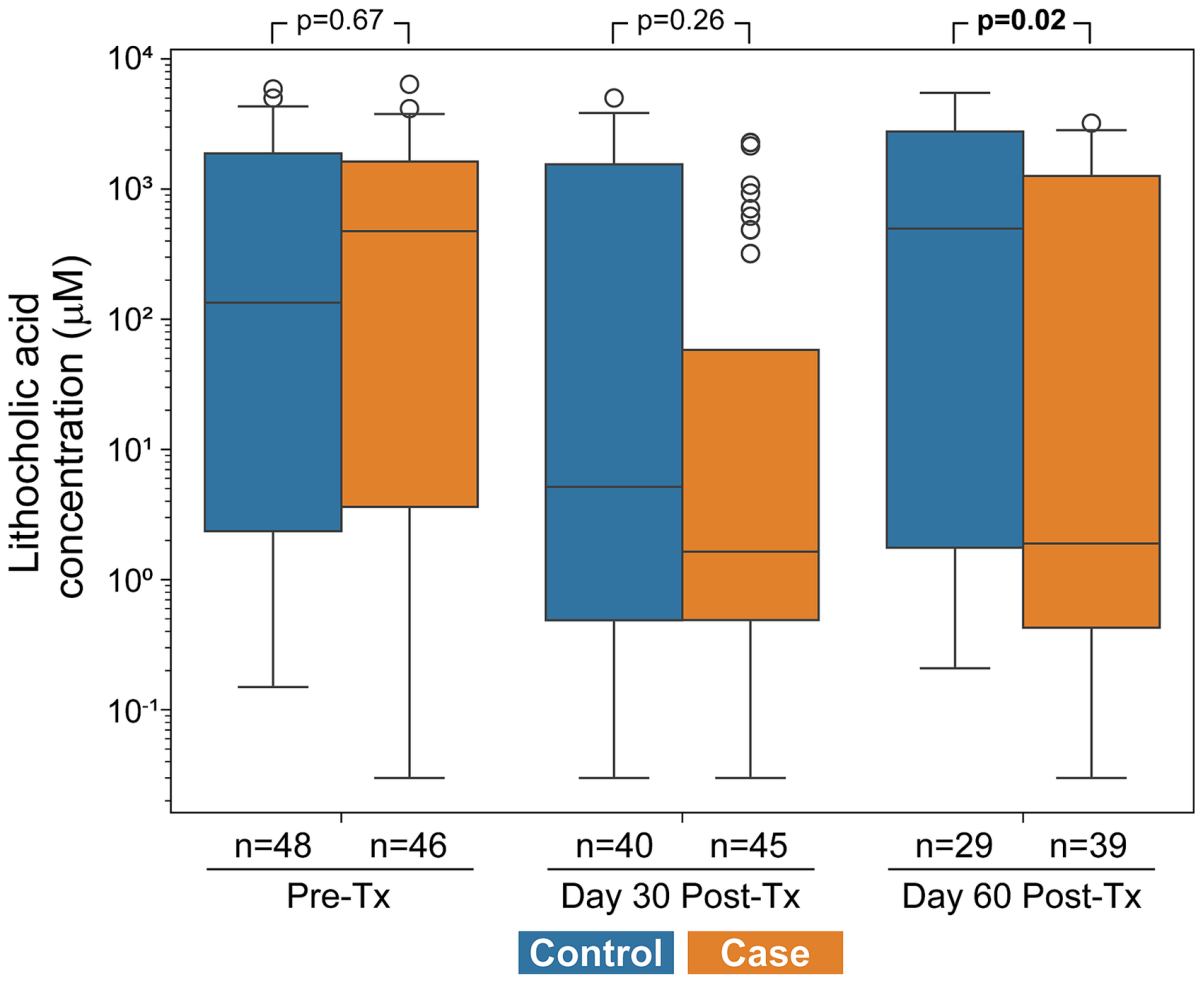
Differences in the endogenous secondary BA, lithocholic acid. There were differences in endogenous secondary BAs 11 between GVHD cases vs. controls, such as seen with lithocholic acid. The bounds of the boxes, the lines within the boxes, the whiskers, and the consideration of outlying values are the same as those defined in Figure 1 legend. The Mann–Whitney U test was performed to generate *p*-values. Tx = hematopoietic cell transplant.

Since significant differences in summed endogenous secondary bile acid levels were observed between GvHD cases vs. controls post-HCT, we also examined each endogenous secondary bile acid (Table 2; Figure 3; Supplementary Tables 3, 4). We noted 263-fold lower levels of the endogenous secondary bile acid lithocholic acid (LCA) in GvHD cases (median 1.9 μM) vs. no-GvHD controls (500 μM) on day 60 post-transplant (*p* = 0.02/p_adj_ = 0.16, FC = 0.004; Figure 3; Supplementary Table 3). Similarly, LCA levels in pre-transplant GvHD cases (median 480 μM) decreased 300-fold to 1.6 μM by day 30 post-transplant (*p* < 0.001, FC = 0.003; Supplementary Figure 3F). Consistent with summed endogenous secondary bile acids as a group, day 60 post-transplant LCA levels in GvHD cases were 250-times reduced from pre-transplant levels (*p* = 0.02, FC = 0.004; Supplementary Figure 3F), which showed the persistent loss of LCA was unique to GvHD (vs. no-GvHD).

**Table 2:** Differences in concentrations of individual endogenous secondary BAs.

Bile acid	Time point	Controls	Cases	Difference	*p*-value	Adjusted *p*-value
Taurolithocholic acid	Day 30	0.6	0.2	0.4	0.013	0.157
Taurolithocholic acid	Day 60	0.4	0.2	0.2	0.053	0.222
5beta-cholanic acid-3beta, 12alpha-diol	Day 60	19.5	4.4	15.1	0.011	0.157
Glycolithocholic acid	Day 30	2.7	1.9	0.8	0.027	0.157
Lithocholic acid	Day 60	497.8	1.9	495.9	0.019	0.157
7KLCAolone*	Day 60	169.4	49.7	119.7	0.035	0.167
12KLCA814*	Day 60	54.3	0.2	54.1	0.029	0.157

Table summarizing significant differences in concentrations of lithocholic acid isomers and derivatives between GvHD cases vs. controls. Difference in medians are calculated by subtracting the case from the control median concentration. Statistical significance and *p*-values for comparing the distribution of concentrations from the Mann–Whitney U test. *p*-Values were adjusted for false discovery rate (FDR). Median concentrations in μM are shown. *7KLCAolone is an abbreviation for the bile acids 3alpha-hydroxy-7-ketolithocholic acid and 5alpha-cholanic acid-3alpha-ol-6-one, which are indistinguishable from each other in our LC–MS analysis. 12KLCA814 is an abbreviation for the bile acids 3alpha-hydroxy-12 ketolithocholic acid and 8(14), (5beta)-cholenic acid-3alpha, 12alpha-diol, which are indistinguishable from each other in our LC–MS analysis.

Six additional endogenous secondary bile acids were less prevalent and/or at lower concentrations in GvHD cases vs. controls post-transplant (Tables 2, 3). Concentrations of tauroLCA and glycoLCA (conjugated forms of LCA) were significantly lower (3 and 1.4-fold, respectively) in GvHD cases vs. controls at 30 days post-transplant (*p* = 0.01/p_adj_ = 0.16 and 0.03/0.16, respectively; FC = 0.33 and 0.70, respectively; Table 2). Concentrations of tauroLCA and LCA were also lower (2 and 263-fold, respectively) in GvHD cases at 60 days post-transplant (*p* = 0.05/p_adj_ = 0.22 and 0.02/0.16, respectively; FC = 0.50 and 0.004, respectively; Table 2). At 30 days post-transplant, alloLCA (LCA conformer) was less prevalent in GvHD cases than controls (*p* = 0.05/p_adj_ = 0.35; Table 3). Bile acids tauroLCA and LCA were also less prevalent in GvHD cases at 60 days post-transplant (*p* = 0.05/p_adj_ = 0.35 and 0.02/0.29, respectively; Table 3). It was striking that five of the significantly different endogenous secondary bile acids were highly structurally similar to LCA.

**Table 3:** Differences in prevalence of individual endogenous secondary BAs.

Bile acid	Time-point	Controls	Cases	Difference	*p*-value	Adjusted *p*-value	Odds ratio	Odds ratio 95% CI
Allolithocholic acid	Day 30	37.50 (15)	17.78 (8)	19.72	0.052	0.354	2.74	0.93–8.68
Taurolithocholic acid	Day 60	86.21 (25)	64.10 (25)	22.11	0.054	0.354	3.44	0.91–16.36
Lithocholic acid	Day 60	100.00 (29)	82.05 (32)	17.5	0.017	0.288	Inf**	1.17–inf**
12KLCA814*	Day 60	68.97 (20)	43.59 (17)	25.38	0.05	0.354	2.83	0.94–9.02
5beta-cholanic acid-3beta, 12alpha-diol	Day 60	96.55 (28)	66.67 (26)	29.88	0.002	0.078	13.58	1.8–613.81

Table summarizing significant differences in prevalence of lithocholic acid isomers and derivatives between GvHD cases vs. controls. Statistical significance and *p*-values for comparing prevalence derived from Fisher’s Exact test. *p*-Values were adjusted for false discovery rate (FDR). Prevalence data are shown in % (count, #). *7KLCAolone is an abbreviation for the bile acids 3alpha-hydroxy-7-ketolithocholic acid and 5alpha-cholanic acid-3alpha-ol-6-one, which are indistinguishable from each other in our LC–MS analysis. 12KLCA814 is an abbreviation for the bile acids 3alpha-hydroxy-12 ketolithocholic acid and 8(14),(5beta)-cholenic acid-3alpha, 12alpha-diol, which are indistinguishable from each other in our LC–MS analysis. **The reported “inf” or “infinite” odds ratio and corresponding 95% confidence intervals are unable to be estimated due to counts of 0 and division by 0 errors. CI = confidence interval.

To delve deeper into the LCA findings, we revisited the day 30 post-HCT GvHD sample classifications (15 cases pre-GvHD, 30 cases with current GvHD, 40 no-GvHD controls). Adjustment or normalization by time to GvHD diagnosis was performed at this time point by comparing 55 samples without GvHD (ever or yet) vs. 30 samples with GvHD. This analysis revealed that concentrations of two LCA-like bile acids, alloLCA and tauroLCA, as well as the sum of the 11 endogenous secondary bile acids, were significantly lower during current GvHD vs. the combined samples without GvHD (*p* = 0.009/p_adj_ = 0.11, 0.006/0.11, and 0.01/0.11, respectively; Supplementary Table 4).

Next, we sought to quantify the effect size of the six bile acids significantly associated with GvHD by calculating the odds ratio for GvHD and corresponding 95% confidence intervals. The odds ratios indicate that the presence of each bile acid in the gastrointestinal tract was associated with a reduced chance of having GvHD. The odds ratios for 5beta-cholanic acid-3beta, 12alpha-diol were the largest at 14 (95% confidence interval [CI] = 1.8–610; Table 3). The odds ratio of LCA is incalculable (“inf”) due to 100% prevalence among 29 control patients without GvHD at 60 days post-HCT (95% CI = 1.17–inf; Table 3). Nonetheless, after adjusting *p*-values for multiple testing (FDR), the adjusted differential concentration and prevalence *p*-values exceeded 0.07 and were no longer statistically significant (Tables 2, 3). Thus, we sought to query an analogous dataset to determine if our findings (particularly the LCA findings) were reproducible.

### 3.5 Analysis of the association between bile acid deficiency and GvHD at another cancer center

We analyzed a publicly available dataset from a comparable cancer center to assess the reproducibility of our findings regarding secondary bile acids and GvHD. These data describe bile acid levels in a patient population that received allogeneic HCT at Memorial Sloan Kettering (MSK) Cancer Center. The data were published by Lindner et al. in 2024 and represent a different patient population and Center than this study. We delved into the supplemental data file and brought the reported concentrations and associated metadata into the Python environment for differential abundance analysis (including Mann–Whitney U test). Several findings were concordant in this dataset; namely, LCA was depleted in GvHD cases vs. controls overall (*p* = 0.0007), and at both time points examined in the Lindner study, peri-engraftment (*p* = 0.02) and peri-GvHD (*p* = 0.02; Supplementary Figure 7). Also of interest was lithocholic acid-3-sulfate (not included in this study’s bile acid metabolomic panel), which was lower in GvHD cases vs. controls overall (*p* = 0.007) and at peri-engraftment (*p* = 0.0004; Supplementary Figure 7) (Lindner et al., 2024). Indeed, the MSK group’s LCA findings were consistent with those observed in this study.

### 3.6 Endogenous bile acid levels remain low in GvHD even when prednisone therapy is considered

We investigated whether prednisone/methylprednisolone therapy could have biased the measured bile acid concentrations in stool samples by excluding the 14 samples that were collected from patients while they were receiving this steroid treatment. Bile acid levels were then re-analyzed in a sub-cohort that was off active prednisone/methylprednisolone therapy. Lithocholic acid and endogenous secondary bile acid levels remained significantly reduced in the participants with vs. without GvHD 60 days post-HCT (*p* = 0.02 and 0.002, respectively; Supplementary Figure 8).

### 3.7 Bile acid-modulating microbes associate with endogenous secondary bile acids post-HCT

**Figure 4:**
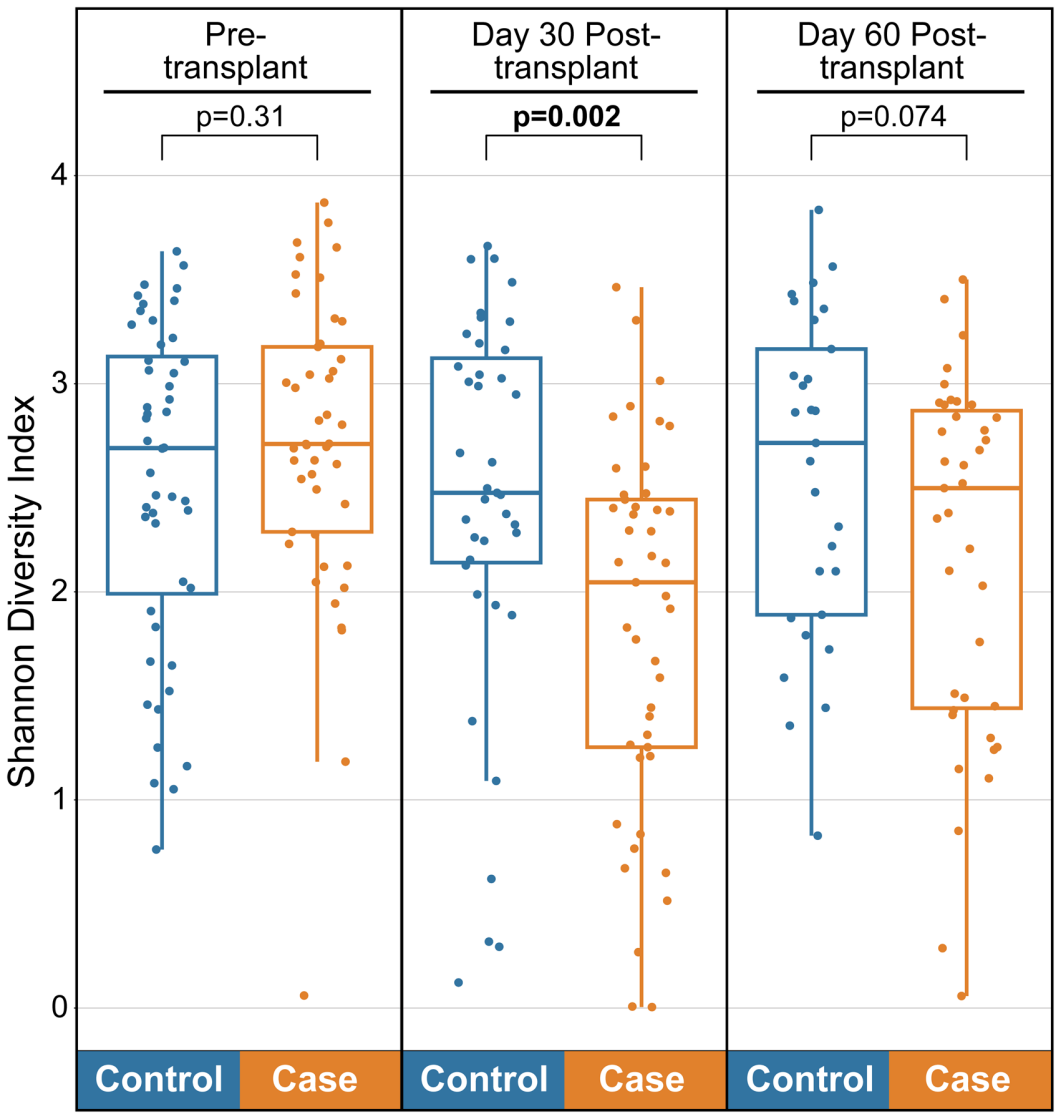
Community-level differences in gut microbial diversity. Microbial alpha-diversity was associated with GvHD at the 30-day post-HCT time point. The bounds of the boxes, the lines within the boxes, the whiskers, and the consideration of outlying values are the same as those defined in Figure 2 legend. The Mann–Whitney U test was performed to generate *p*-values.

With evidence that our study’s LCA findings were reproducible, the next question was: why are LCA and LCA-like endogenous secondary bile acids deficient during GvHD? It is known that certain gut microbes metabolize bile acids (Supplementary Figure 1). Therefore, we hypothesized that differences in GvHD case vs. control gut microbial communities may account for differences in secondary bile acid levels. We first investigated bacterial community-level differences between GvHD cases and controls by comparing alpha-diversity as measured by the Shannon Diversity Index (SDI) using 16S rRNA gene sequencing data from stool samples (Figure 4). SDI in this context is a metric of bacterial species richness (number of species) and evenness in stool samples By 30 days post-transplant, the median Shannon Diversity Index (SDI) in cases was 2.05, which was significantly lower than that of no-GvHD samples, 2.49 (*p* = 0.002; Figure 4). At 60 days post-transplant, the median SDI was 2.50 in GvHD cases and 2.72 in controls (*p* = 0.074; Figure 4). These data provide evidence that gut bacterial diversity is lower post-HCT in patients with GvHD in our study.

We next sought to determine how this difference in alpha-diversity and stool bacterial composition might lead to differences in bile acid concentrations. We measured bacterial relative abundance and prevalence at each time point using 16S rRNA gene PCR with sequencing, but no specific taxa at the family, genus or species level was statistically significantly different by GvHD status when using the Mann Whitney U test and when adjusting for multiple comparison (Supplementary Figure 9). Despite this, we found that numerous microbial taxa correlated with concentrations of endogenous secondary bile acids (Table 4). Notably, *Clostridium scindens* (a *bai*-possessing microbe) associated with the LCA-like endogenous secondary bile acid 3alpha-hydroxy-12 ketolithocholic acid/8(14),(5beta)-cholenic acid-3alpha, 12alpha-diol (12KLCA814) at 30 days post-HCT (correlation coefficient rho = 0.57, p_adj_ = 1.87E−6). *Dysosmobacter welbionis* associated with the LCA conformer, alloLCA (rho = 0.57, p_adj_ = 4.34E−6; Table 4), at 60 days post-HCT. AlloLCA also correlated with multiple microbial taxa: *Ruminococcus torques* at 60 days post-HCT (rho = 0.61, p_adj_ = 6.49E−7; Table 4), *Lachnospiraceae* on day 60 post-HCT (rho = 0.59, p_adj_ = 1.28E−6), and *Oscillospiraceae* on day 30 post-HCT (rho = 0.58, p_adj_ = 2.51E−6; Table 4). Further, we noticed that these taxa have a key commonality.

**Table 4:** Bile acids and microbial taxa correlate post-HCT.

Bile acid	Microbial taxa	rho	*p*-value	Adjusted *p*-value	Time point
12KLCA814	*Clostridium scindens*	0.57	2.53E−09	1.87E−06	Day 30
Deoxycholic acid	*Clostridium scindens*	0.55	4.70E−08	1.67E−05	Day 60
3-ketocholanic acid	*Ruminococcus torques*	0.59	2.46E−10	3.39E−07	Day 60
Allolithocholic acid	*Ruminococcus torques*	0.61	7.22E−10	6.49E−07	Day 60
Allolithocholic acid	*Oscillospiraceae*	0.58	3.96E−09	2.51E−06	Day 30
3-ketocholanic acid	*Dysosmobacter welbionis*	0.57	8.26E−10	6.49E−07	Day 60
Allolithocholic acid	*Dysosmobacter welbionis*	0.57	7.89E−09	4.34E−06	Day 60
3-ketocholanic acid	*Lachnospiraceae*	0.56	6.42E−10	6.49E−07	Day 60
Allolithocholic acid	*Lachnospiraceae*	0.59	1.86E−09	1.28E−06	Day 60
Allolithocholic acid	*Neglecta*	0.65	1.77E−10	3.25E−07	Day 60
Allolithocholic acid	*Neglecta*	0.55	1.20E−08	3.54E−06	Day 30
3-ketocholanic acid	*Eubacteriales*	0.63	7.30E−12	2.01E−08	Day 60
3-ketocholanic acid	*Eubacteriales*	0.55	7.35E−11	3.27E−07	Day 30
Allolithocholic acid	*Eubacteriales*	0.76	5.15E−15	2.83E−11	Day 60
Allolithocholic acid	*Eubacteriales*	0.57	6.80E−10	1.01E−06	Day 30

Correlations between abundance of microbial taxa and bile acids that were moderately strong (rho ≥ 0.55). Several bile acids link with multiple microbial taxa, and vice versa. These strong correlations emerge post-HCT. Kendall Tau correlation was used to evaluate the relationships between bile acids and 16S microbial taxa. ‘Time point’ refers to the time point post-transplant.

Several microbial taxa in Table 4 that moderately strongly correlated with a bile acid (in this study, a moderately strong correlation coefficient rho was considered ≥0.55) either possess or include members that possess *bsh* (e.g., *Oscillospiraceae, Lachnospiraceae, D. welbionis, R. torques*) and/or *bai* genes (e.g., *C. scindens*), but it is challenging to assess bile acid metabolism by taxonomy alone (Nucleotide [Internet], 2021; Nucleotide [Internet], 2024; Nucleotide [Internet], 2019; Nucleotide [Internet], 2022; Nucleotide [Internet], 2015; Nucleotide [Internet], 2017). This is because *bsh* genes are highly diverse in sequence, making it difficult to use sequence-based methods (such as PCR) to characterize them. *Bsh* gene possession is also highly inconsistent and yet abundant, with *bsh* genes present at zero to four copies per genome, even within a single taxon, such as a given species or strain (Guzior et al., 2024; Song et al., 2019; Jones et al., 2008; Daly et al., 2021; O’Flaherty et al., 2018; Rimal et al., 2024). Gene orthologs of *bsh* within the same genome or taxon may not have high sequence similarity, similar functions, or target specificity/efficiency (Song et al., 2019; Lambert et al., 2008; Liang et al., 2018; Franz et al., 2001; Tanaka et al., 2000; Begley et al., 2005). Conversely, the presence of the *bai* gene is more conserved within a species. Altogether, this informed our decision to adopt a gene-forward approach to further probe microbial bile acid metabolism.

### 3.8 Loss of secondary bile acid-producing *bai* genes associates with GvHD post-HCT

**Figure 5:**
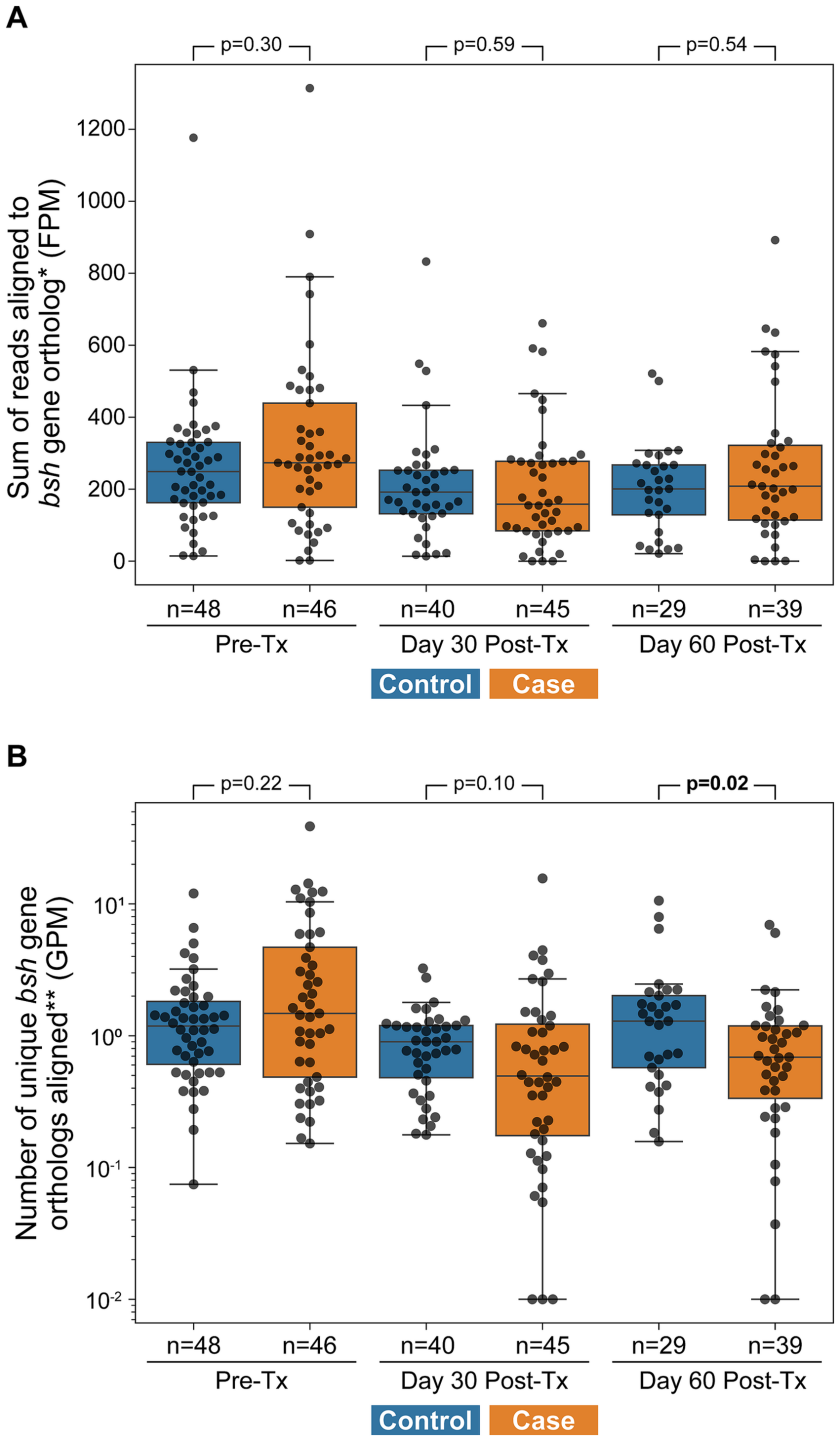
Abundance and diversity of deconjugation *bsh* genes. **(A)** The distributions of the sum of reads that aligned to a *bsh* gene ortholog (normalized to the sum of bacterial reads per sample) in a sample were statistically the same between GvHD cases and controls at each time point. *Bsh reads were normalized to the sum of bacterial reads per sample, reported in fragments per million (FPM). **(B)** Metagenomic reads aligned to statistically fewer *bsh* gene orthologs (normalized to the sum of bacterial reads per sample) in GvHD cases vs. controls at day 60 post-transplant. At pre-transplant and 30 days post-transplant, GvHD cases and controls had statistically similar numbers of *bsh* genes hit by the metagenomic reads. **Number of *bsh* genes was normalized to the sum of bacterial reads per sample, reported in genes per million (GPM). A read cutoff of 5 was implemented to consider any genes a true hit. The bounds of the boxes, the lines within the boxes, the whiskers, and the consideration of outlying values are the same as those defined in Figure 1 legend. The Mann–Whitney U test was performed to generate p-values.

**Figure 6:**
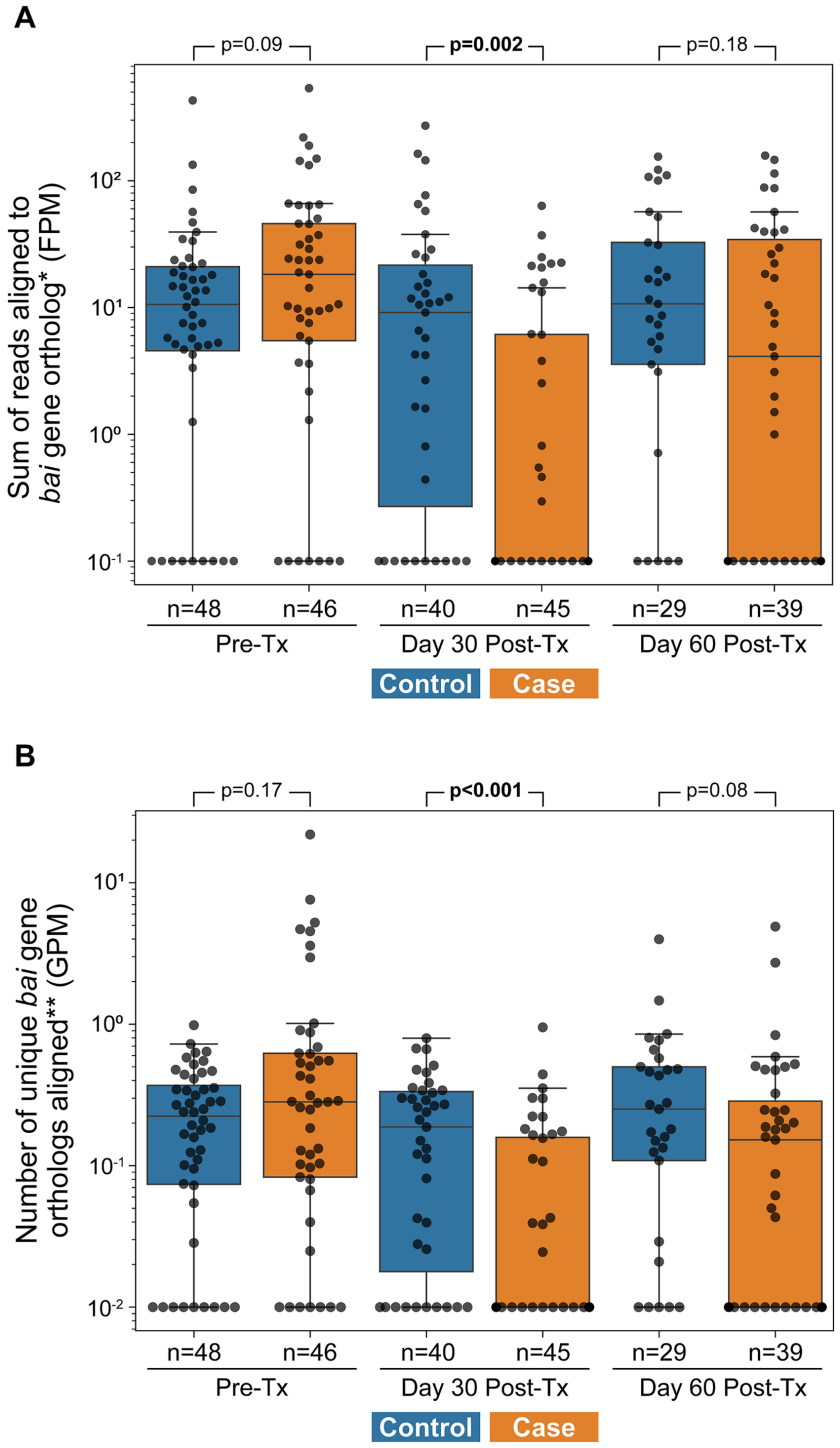
Abundance and diversity of 7α-dehydroxylation *bai* genes. **(A)** The sum of reads that aligned to a *bai* gene ortholog (normalized to the sum of bacterial reads per sample) in a sample was statistically significantly greater in controls than in GvHD cases at 30 days post-transplant. Pre-transplant, the (normalized) sums of reads aligned to a *bai* gene ortholog were higher in GvHD cases than in controls, although this was not statistically significant. There was no statistical difference in the sum of reads aligned to a *bai* gene at 60 days post-transplant based on GvHD status. *Bai reads were normalized to the sum of bacterial reads per sample, reported in fragments per million (FPM). **(B)** Metagenomic reads aligned to statistically fewer *bai* gene orthologs (normalized to the sum of bacterial reads per sample) in GvHD cases vs. controls at day 30 post-transplant. 60 days post-transplant, GvHD cases appeared to have fewer *bai* gene orthologs hit than controls, although this was not statistically significant. Pre-transplant, GvHD cases and controls had statistically similar numbers of *bai* genes hit by the metagenomic reads. **The number of *bai* genes was normalized to the sum of bacterial reads per sample, reported in genes per million (GPM). A read cutoff of 5 was implemented to consider any genes a true hit. The bounds of the boxes, the lines within the boxes, the whiskers, and the consideration of outlying values are the same as those defined in Figure 1 legend. The Mann–Whitney U test was performed to generate *p*-values.

The gene-forward approach involved a targeted *bsh* and *bai* gene survey in stool, performed for GvHD case and control samples using shotgun metagenomic sequencing data. This method quantified capacity for bile acid metabolism in gut microbial communities without using taxonomy as a proxy. Surprisingly, the normalized sum of metagenomic reads that aligned to a *bsh* gene ortholog did not associate with GvHD status at any time point (*p* values > 0.30; Figure 5A). Rather, a greater number of unique *bsh* gene orthologs associated with absence of GvHD at day 60 post-HCT (*p* = 0.02, FC = 3.2; Figure 5B). This was concordant with the observed association between a diverse microbial community and absence of GvHD (Figure 4) and suggests that quantity of bsh gene orthologs may be less important than type(s) or diversity of *bsh* gene orthologs. For the *bai* genes, a 100-fold lower normalized sum of reads that aligned to *bai* gene orthologs associated with GvHD 30 days post-allo-HCT (*p* = 0.002, FC < 0.01, the day 30 post-HCT median sum of *bai* reads in GvHD cases is the pseudocount 0.1; Figure 6A). Diversity of *bai* genes was also important as a 20-fold lower number of unique *bai* gene orthologs associated with GvHD 30 days post-allo-HCT (*p* = 0.0007, FC < 0.05, the day 30 post-HCT median number of *bai* genes in GvHD cases is the pseudocount 0.01; Figure 6B). Finally, detection of at least one *bai* gene ortholog was associated with absence of GvHD 30 days post -HCST (*p* = 0.004/p_adj_ = 0.01, odds ratio: 4.1; Table 5), further highlighting the importance of *bai* genes.

**Table 5:** Prevalence of 7α-dehydroxylation *bai* genes.

Time point	GvHD status	Number of samples with the *bai* gene ortholog present	Total number of samples	Prevalence (%)	*p*-value	Adjusted *p*-value	Odds ratio	Odds ratio 95% CI
Pre-transplant	Case	38	45	84.44	0.786	0.786	0.8	0.23–2.70
	Control	39	48	81.25				
Day 30	Case	18	44	40.91	**0.004**	**0.011**	4.112	1.50–12.01
	Control	29	39	74.36				
Day 60	Case	24	39	61.54	0.066	0.099	2.953	0.85–12.09
	Control	24	29	82.76				

At least one *bai* gene ortholog was detected in a greater portion of GvHD case samples vs. no-GvHD samples at days 30 and 60 post-transplant. Pre-transplant, at least one *bai* gene ortholog was detected in the same portion of GvHD case and control samples. A read cutoff of 5 was implemented to consider any genes a true hit. Bold values denote statistically significant *p*-values (≤ 0.05). Fisher’s Exact test was performed to generate *p*-values. *p*-Values are FDR-adjusted.

## 4 Discussion

Despite human leukocyte antigen matching to reduce GvHD post-HCT, many HCT recipients still develop GvHD (Arai et al., 2015; Storb et al., 2013). Why there is such heterogeneity in GvHD development/recovery after HCT, and the environmental factors and mechanisms that contribute have yet to be determined. It is known that the gut microbiome is associated with GvHD (Staffas et al., 2017; Andermann et al., 2018; Shono and van den Brink, 2018). There are many potential mechanisms by which the gut microbiota may influence GvHD, including through impacts on immune cells, epithelial cells, and the gut barrier; bile acids are known to affect all of these. In this study, we found a reduction in the summed endogenous secondary bile acid concentrations in stool from GvHD cases post-HCT. All HCT recipients are given an exogenous secondary bile acid, UDCA (ursodiol), to prevent liver complications after HCT. Furthermore, six out of 11 endogenous secondary bile acid concentrations measured by the targeted panel in our study were decreased in GvHD cases post-HCT compared to controls. Each of these endogenous secondary bile acids has a structure similar to that of lithocholic acid. Four of these endogenous secondary bile acids are also less prevalent in GvHD vs. no-GvHD controls. Allolithocholic acid is an additional endogenous secondary bile acid that is less prevalent in GvHD. Some studies posit that UDCA administration increases LCA levels in blood, yet all participants in the current study received UDCA, and we observed differences in LCA levels in stool (Sinakos et al., 2010).

As expected, all patients lose some microbial diversity due to the antibiotics administered to every patient. This is reflected in dynamic changes in each patient’s microbial community function, such as the *bai* gene operon that affects microbial bile acid metabolism and concentrations. However, this dynamic/variance is more pronounced in GvHD cases than in non-GvHD controls, prompting the question: why are endogenous secondary bile acid concentrations lower in patients with GvHD than in controls? This may be due to a reduction of microbial *bai* genes and *bai* gene diversity in stool from HCT recipients with GvHD, reflecting loss of 7α-dehydroxylase activity needed to produce secondary bile acids. Metagenomic analysis is a more useful indicator of the presence of *bai* or *bsh* genes in stool than microbial taxonomy. We noted that the absence of *bai* genes in stool samples is associated with GvHD. In contrast, *bsh* genes were detected in every stool sample in our study. This suggests the *bai-*mediated step (not the *bsh-*mediated step) may be rate-limiting in the production of secondary bile acids in these transplant recipients. Additional support for this observation includes the lack of any significant difference in the summed levels of *bsh*-modified bile acids (primary and conjugated) comparing cases to controls. *Bai* gene products are exclusively responsible for the 7α-dehydroxylation (*bai*) step that produces secondary bile acids. Host genetic and other factors do not contribute to secondary bile acid production. HCT recipients with GvHD may not have sufficient *bai-*possessing microbes. This is reflected by: (A) lower microbial diversity, as observed in our 16S rRNA gene sequencing analysis, and (B) reductions in both the abundance and diversity of *bai* genes encoding enzymes that produce endogenous secondary bile acids, as reflected in our metagenomic analysis. For example, *Clostridium scindens* encodes *bai* genes and is associated with levels of individual endogenous secondary bile acids. This example supports the potential involvement of *bai-*possessing microbes in the observed endogenous secondary bile acid and *bai* gene depletion. *Bsh* was found in every stool sample from patients with and without GvHD, but there was greater *bsh* gene diversity in controls 60 days post-HCT. This observation suggests that having a diverse set of *bsh* enzymes may be more optimal for converting conjugated bile acids into primary bile acids, and then on to secondary bile acids.

A strength of this time analysis study is the inclusion of both pre- and post-HCT samples to compare microbiome dynamics and bile acid concentrations over time, but the cadence of sample collection does not distinguish among endogenous secondary bile acids as simply markers of GvHD, or within the pathway to GvHD, or both. Pre-transplant bile acid concentrations did not predict GvHD; by day 30 post-transplant, 30 of 45 patients had developed GvHD. The distribution of bile acids at 30 days post-HCT is described in Supplementary Figure 6 and Supplementary Table 4, where endogenous secondary bile acid levels are lower in the 30 patients with GvHD vs. (a) the 15 patients without GvHD yet and (b) the no GvHD controls. It is possible that changes in endogenous secondary bile acids precede GvHD onset by days or weeks, and a monthly sampling frequency would not capture that change. Alternatively, changes in secondary bile acids may be concordant with the onset of GvHD and thus serve as a marker rather than a predictor, while still contributing to the maintenance of GvHD. One way to determine if secondary bile acids contribute to the induction or maintenance of GvHD would be to administer these bile acids or similar bile acid receptor agonists in human or animal studies to assess the impact on GvHD.

How might endogenous secondary bile acids contribute to GvHD? Bile acids can signal through receptors on IECs and immune cells (Wang et al., 1999; Kawamata et al., 2003). This signaling could modulate a range of pleiomorphic host responses, like bile acid production/release, metabolic activity, and immunity (Paik et al., 2022; Hang et al., 2019; Campbell et al., 2020; Guo et al., 2016; Haselow et al., 2013; Perino et al., 2014). Of particular interest is immune cell activation because GvHD is mediated by T cells. Bile acids 3-oxolithocholic acid and isoallolithocholic acid inhibit T-helper type 17 cells and promote T regulatory cell differentiation in mice (Paik et al., 2022; Hang et al., 2019; Campbell et al., 2020). Bile acids can also bind cell membrane receptor TGR5 (LCA > DCA > CDCA), present on macrophages and dendritic cells, and nuclear membrane receptor Farnesoid X receptor (FXR; CDCA > DCA > LCA > CA > UDCA), present on T cells, natural killer (NK) cells, NKT cells, and myeloid cells such as neutrophils, macrophages and dendritic cells (Ding et al., 2015; Wang et al., 1999; Kawamata et al., 2003; Campbell et al., 2020; Vavassori et al., 2009; Jones et al., 2023). These connections are highly relevant because Th17 cells are a main driver of GvHD, whereas Treg cells limit T cell activation and thus GvHD, providing a plausible, direct pathway for bile acids to modulate GvHD (Yi et al., 2009; Gartlan et al., 2017; Bettelli et al., 2006; Edinger et al., 2003; Gagliani et al., 2015). Vitamin D receptor (VDR) is another receptor of interest that binds LCA but no other bile acids (Makishima et al., 2002). VDR is present on IECs and T cells, both of which are crucial for GvHD, as IEC-mediated antigen presentation specifically drives the priming and activation of donor T cells that initiate GvHD (Koyama and Hill, 2019; Koyama et al., 2019). VDR activity, like that which can be triggered by LCA binding and activation, can promote tight junction protein gene expression to preserve intestinal barrier integrity, abrogate pro-inflammatory IL-12, NFκB, and TNFα gene expression, and impact differentiation of T cells (Rosenblatt et al., 2010; Kong et al., 2008; Kongsbak et al., 2013; D’Ambrosio et al., 1998). Vitamin D deficiency has been extensively studied in the context of GvHD and linked to poor overall survival post-HCT in humans (Arain and Matthiesen, 2019; Silva et al., 2011). Case reports describe the resolution of chronic GvHD symptoms and reduced relapse rates post-HCT in human recipients supplemented with Vitamin D (Yigenoglu et al., 2025). Since TGR5 is also present in macrophages and dendritic cells, bile acids can influence these cell subsets that present antigens and indirectly contribute to GvHD by promoting T cell activation (Vavassori et al., 2009; Gadaleta et al., 2011; Fiorucci et al., 2018; Penna and Adorini, 2000; Griffin et al., 2001; Piemonti et al., 2000). Additional immunomodulatory effects of bile acids include downregulation of proinflammatory genes and activation of the inflammasome, where the immune cell type involved (e.g., T cells vs. macrophages) and context determine the exact impact on gastrointestinal immune regulation (Fiorucci et al., 2022). An alternative pathway draws on bile acids’ amphipathic properties, permitting their breakdown of dietary fats and their antimicrobial effects. Endogenous secondary bile acids may solubilize bacterial phospholipid outer membranes, reducing the risk of intestinal pathogen growth that would contribute to inflammation and exacerbate GvHD. LCA, an endogenous secondary bile acid, is the most hydrophobic bile acid (LCA > DCA > CDCA > CA > UDCA). LCA exhibits antibacterial activity against GvHD-associated bacteria like *Enterococcus, Staphylococcus, Escherichia,* and others (McKenney et al., 2019; do Nascimento et al., 2015). Thus, LCA’s observed deficiency could permit proliferation of such bacteria to exacerbate GvHD. However, one challenge with using lithocholic acid as an agonist is that high concentrations in the gut can be pro-inflammatory (Cai et al., 2017).

Key findings in our study were concordant with findings from another human study by Lindner et al., published in 2024 (Lindner et al., 2024). This includes deficiencies of LCA, endogenous secondary bile acids, and the sum of *bai* operon genes in GvHD cases vs. controls. Conversely, summed bile acid levels were reported as lower in GvHD cases than in control samples in the Lindner study, but not in the current study. This difference may reflect different study designs. In Lindner et al., key time points of interest were chosen based on individual diagnosis of GvHD (e.g., pre-GvHD onset, peri-GvHD onset). Our study used absolute time to establish time points of interest. Thus, the time between sample collection/analysis and GvHD differs in the two studies. The former retrospective approach using individual diagnoses of GvHD may better capture the biology of GvHD in an individual patient. Ten additional findings from our study were also consistent with the data by Lindner et al. (2024). This demonstrates our study’s findings are not isolated to a single population or center. Although findings were largely concordant, we recognize that each cohort has a unique exposome, including antibiotics, and this may explain differences between these studies.

Steroid therapy may impact the findings described in this study by promoting bile acid recycling, which in turn decreases intestinal bile acid levels (Out et al., 2014). Bile acids are primarily reabsorbed by small intestine epithelial cells, but IEC damage and sloughing are characteristic of the conditioning that transplant recipients undergo before transplant. Furthermore, damage induced by GvHD at the mucosa may reduce ASBT transporters and thus impair bile acid reabsorption, increasing bile acid levels detected in stool. When we excluded samples collected from patients during active prednisone/methylprednisolone therapy, the significant deficiency in endogenous bile acids, such as lithocholic acid, during GvHD was still observed. This supports that steroid therapy does not bias the fecal bile acid concentrations measured in this study. To the best of our knowledge, this represents a novel contribution to the field, as previous studies characterizing bile acid dynamics in stool post-HCT do not address the effect of steroid therapy on bile acid concentrations. However, this is consistent with reports from the current literature, which support that bile acid levels in stool are unaffected by prednisone therapy (Esparteiro et al., 2024).

There are some limitations to this study. Antibiotics may affect the gut microbiota and bile acid metabolism. All patients in this study received prophylactic, empiric, or directed antibiotic therapy, so there was no unexposed group. Several factors may alter the gut microbiota and therefore bile acids, such as antibiotics and diet; a future analysis will focus on the relationships between specific antibiotics and changes in the gut microbiota and bile acid levels in stool. Microbial RNA was not sequenced, so the *bsh* or *bai* gene expression was uncharacterized. Participants’ stool samples were collected via swab. This facilitated sample collection but prevented reliable measurement of stool weight. Thus, the input stool quantity for LC–MS, 16S rRNA gene sequencing, and metagenomic sequencing was non-normalized. However, the same swabs were used for all participants to ensure consistent collection. The swabs typically contain 50–100 μL of stool. Stool sampling cadence is also limited. Since sampling was conducted approximately monthly, it is unclear how endogenous secondary bile acid levels changed between time points. Higher-frequency sampling (daily or weekly) is necessary to characterize endogenous secondary bile acids dynamically at greater resolution and determine whether changes occur before or after GvHD.

The largest biological difference between GvHD cases and controls is in LCA, with a 260-fold difference between the median GvHD case and control concentrations at 60 days post-HCT, and lower concentrations in GvHD. Additionally, lithocholic acid is present in 100% of controls vs. 82% in GvHD cases on day 60 post-HCT. Thus, LCA derivatives and *bai* genes are promising for further studies examining their possible role as drivers or markers of GvHD. The secondary bile acid UDCA is currently administered to all allo-HCT recipients. UDCA was administered therapeutically to humans as early as 1977, although it was not tested in an animal model until 1985 (Nakagawa et al., 1977; Kitani et al., 1985). It was also used to treat liver GvHD as early as 1992, despite not testing UDCA’s impact on GvHD in an animal model before human use (Fried et al., 1992). Cholic acid (Cholbam), chenodeoxycholic acid (chenodiol/Ctexli), and obeticholic acid (Ocaliva) are other examples of FDA-approved bile acid therapies for humans, providing a precedent for prescribing bile acids to transplant recipients as prophylaxis against adverse transplant outcomes. The data described in this study are in the early pre-clinical stages, but if there is a relationship with GvHD onset or maintenance, then novel therapeutic interventions may be feasible to mitigate gut GvHD. These interventions could include providing *bai* gene-possessing bacteria, such as *C. scindens*, to HCT recipients as a live biotherapeutic product. Another therapeutic option would be to directly administer endogenous secondary bile acids, such as LCA, to HCT recipients post-transplant. This may entail administering a delayed/time-released formulation. Detailed *in vitro* and *in vivo* studies are required to study this concept, since LCA has been observed to have toxic and carcinogenic effects in animal models (Cai et al., 2017; Baek et al., 2010). Less toxic LCA congeners should be considered for GvHD therapy. Well-tolerated alternatives warrant further investigation. Examples include: LCA-sulfate due to its structural similarity to native LCA and cholic acid-7-sulfate due to its agonism of the same receptor as LCA, TGR5 (Ito et al., 2024; Chaudhari et al., 2021).

In summary, we propose that GvHD may be impacted by alterations in gut microbial bile acid metabolism. We showed that bile acid concentrations differ in HCT recipients with and without GvHD. Our study provides evidence that secondary bile acids, especially lithocholic acid, are reduced during GvHD. The deficiency may result from a loss of the gut microbes that possess *bai* genes and produce secondary bile acids, which can influence gut immunity in the setting of GvHD. These findings are summarized in a visual abstract (Supplementary Figure 10). If bile acids contribute to the pathogenesis of GvHD, then lithocholic acid and the cell receptors that bind it (VDR, TGR5) could represent a novel therapeutic axis to explore for the prevention and treatment of GvHD.

## Data Availability

The metabolomics data presented in this study can be found in the Metabolomics Workbench (Sud et al., 2016), project doi: 10.21228/M8HV86. The raw sequencing generated can be found in the NCBI BioProject, accession number PRJNA1479378. Further inquiries can be directed to the corresponding author.

## Footnotes

1
https://www.ncbi.nlm.nih.gov/protein/

2
blast.ncbi.nlm.nih.gov/Blast.cgi

3
github.com/FredHutch/gig-map
