# Supplementary material for: Acute gastrointestinal graft-versus-host disease is associated with reductions of secondary bile acids following allogeneic hematopoietic cell transplantation
Source: Front Microbiol. 2026 Jul 9;17:1818647. doi: 10.3389/fmicb.2026.1818647 (PMC13391574; doi:10.3389/fmicb.2026.1818647)
Supplement: Supplementary file 1 [file Data_Sheet_1.PDF]

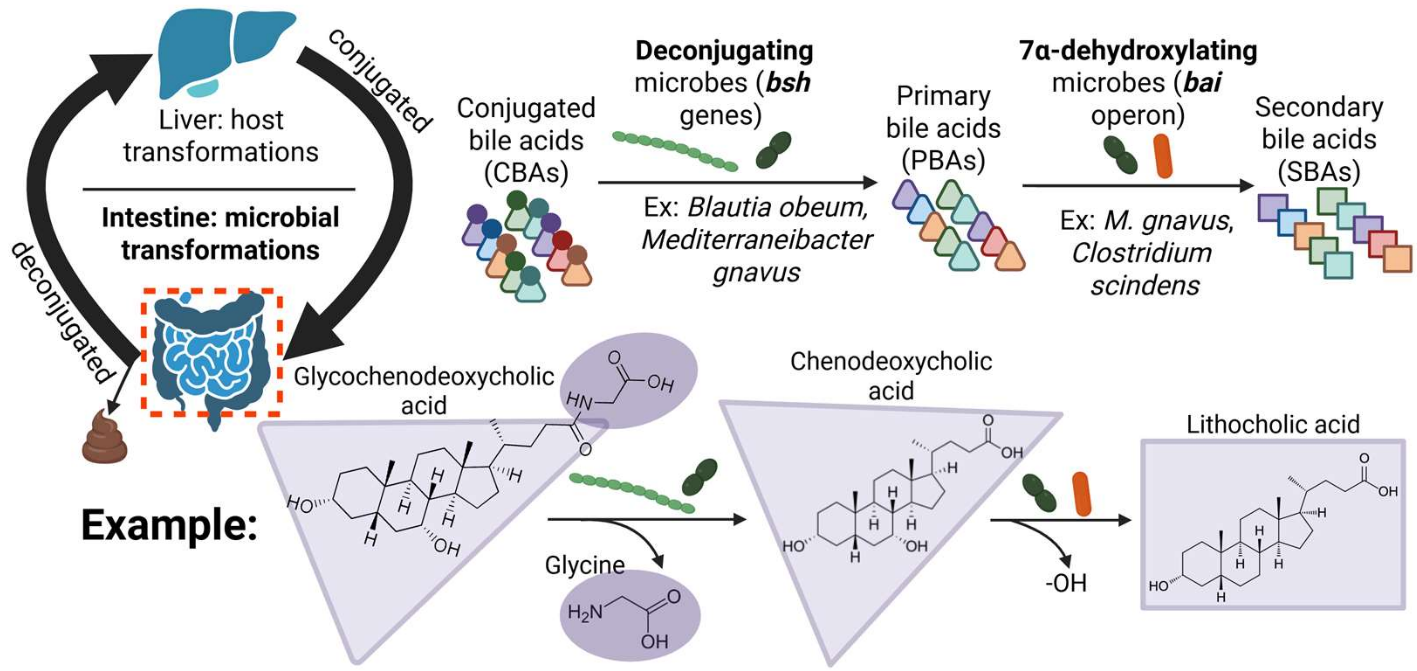

Supplemental Figure 1. Enterohepatic circulation of bile acids. Bile acids are produced from cholesterol in the liver by the host. They undergo several host-mediated biotransformations in the liver before being transported to the intestine as primary bile acids conjugated to taurine or glycine, such as glycochenodeoxycholic acid. Once in the intestine, the taurine or glycine are removed by microbes that possess a *bile salt hydrolase (bsh)* gene encoding a choloylglycine hydrolase enzyme. This process is called deconjugation, after which the bile acids are in primary form, for example, chenodeoxycholic acid. These primary bile acids travel along the intestine and encounter a different subset of microbes that possess the *bile acid inducible (bai)* operon. These microbes can perform 7α-dehydroxylation, removing the hydroxyl group from the seventh carbon within the bile acid structure. This multi-step reaction results in production of secondary bile acids, like lithocholic acid. The majority of intestinal bile acids are reabsorbed and recirculated back to the liver via the portal vein for enterohepatic circulation. A small portion of bile acids are excreted in stool. Created in BioRender. Cagle, R. (2026) https://BioRender.com/wkbvayg.

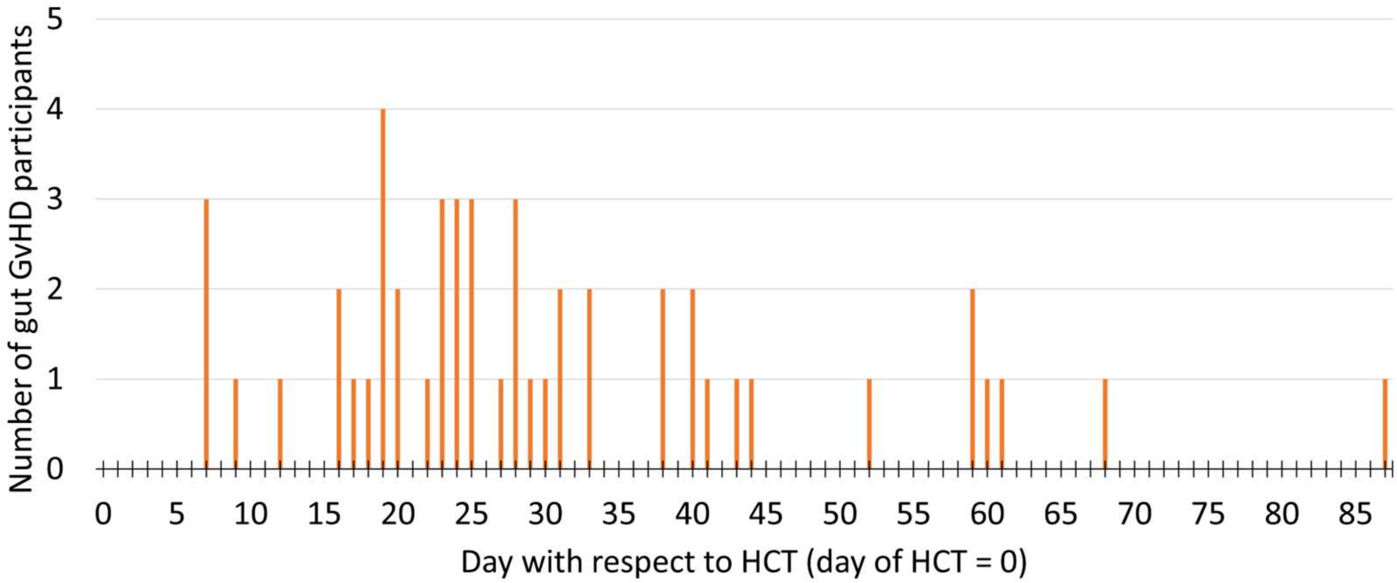

Supplemental Figure 2. Histogram describing the distribution of time to GvHD diagnosis in days relative to HCT (day 0) for all 49 gut GvHD participants. Median time (days) to GvHD is 25.

Concentration (μM)	Compound name	Compound abbreviation	Source
9.94	Deuterated glycochenodeoxycholic acid	d4-GCDCA	Cambridge Isotope Labs (Tewksbury, MA)
9.94	Deuterated lithocholic acid	d4-LCA	Cambridge Isotope Labs (Tewksbury, MA)
9.94	Deuterated deoxycholic acid	d4-DCA	Cambridge Isotope Labs (Tewksbury, MA)
9.94	Deuterated glycocholic acid	d4-GCA	Cambridge Isotope Labs (Tewksbury, MA)
10.08	Deuterated cholic acid	d4-CA	Cambridge Isotope Labs (Tewksbury, MA)

Supplemental Table 1. Composition of Internal Standard stock solution used in targeted bile acid liquid chromatography-mass spectrometry. These compounds are in solvent composed of MeOH and water in a 1:1 ratio.

		Cases (n = 49) (%)	Controls (n = 49) (%)
**Grade acute GvHD**	**0**	0 (0)	34 (69.39)
	**1**	0 (0)	4 (8.16)
	**2**	0 (0)	11 (22.45)
	**3**	35 (71.43)	0 (0)
	**4**	14 (28.57)	0 (0)
**Stage acute skin GvHD**	**0**	28 (57.14)	34 (69.39)
	**1**	1 (2.04)	2 (4.08)
	**2**	8 (16.33)	2 (4.08)
	**3**	9 (18.37)	11 (22.45)
	**4**	2 (4.08)	0 (0)
	**NA**	1 (2.04)	0 (0)
**Stage acute liver GvHD**	**0**	39 (79.59)	49 (100)
	**1**	1 (2.04)	0 (0)
	**2**	5 (10.2)	0 (0)
	**3**	3 (6.12)	0 (0)
	**4**	1 (2.04)	0 (0)
**Stage acute gut GvHD**	**0**	0 (0)	49 (100)
	**1**	0* (0*)	0* (0*)
	**2**	22 (44.9)	0 (0)
	**3**	15 (30.61)	0 (0)
	**4**	12 (24.49)	0 (0)

Supplemental Table 2. GvHD demographics of the patient population. *Participants with stage 1 acute gut GvHD were excluded from study cohort to focus on differences between no acute gut GvHD (stage 0) and moderate-severe acute gut GvHD (stages 2-4)

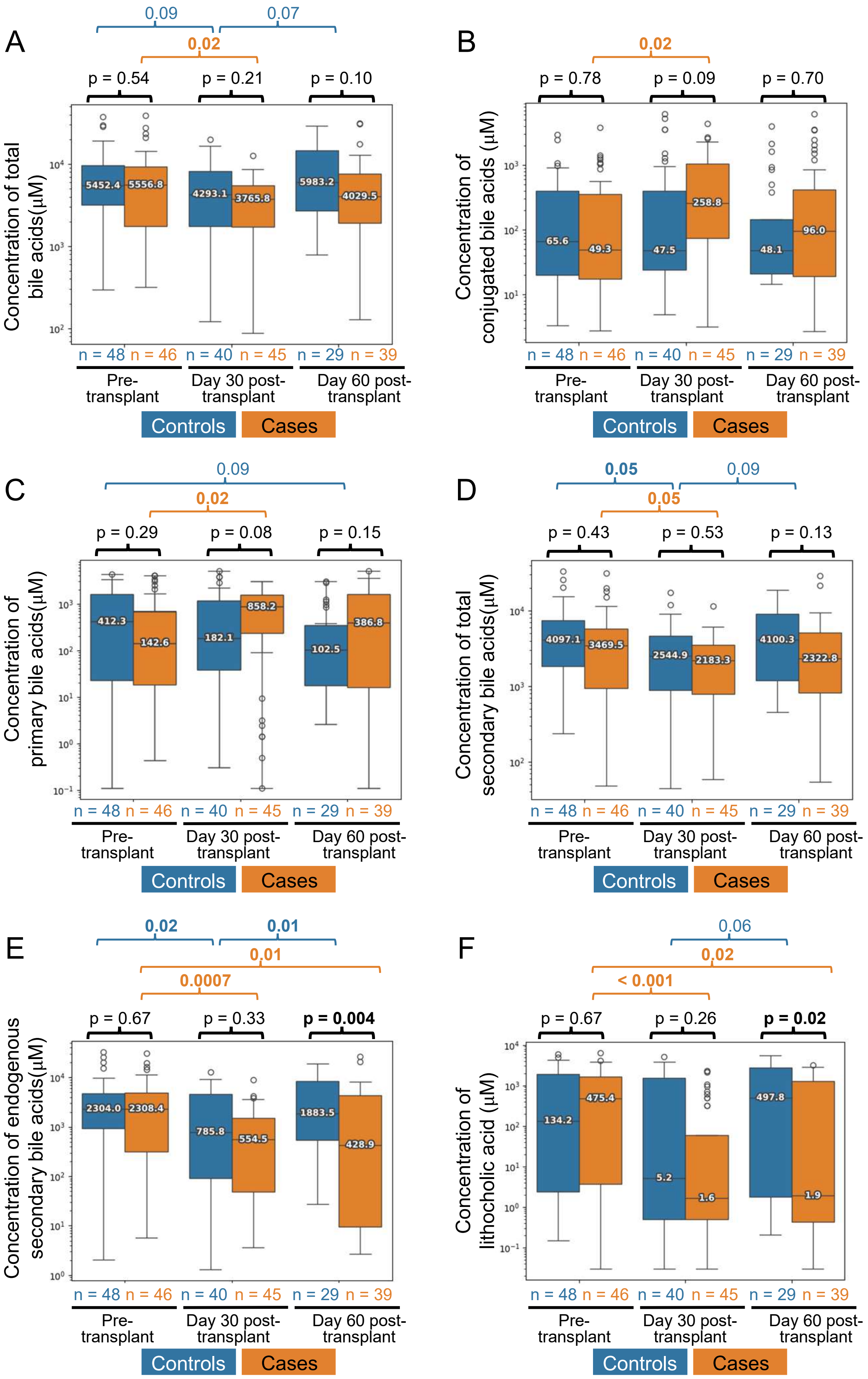

Supplemental Figure 3. Differences in bile acid levels of within-group changes over time. (**A**) Total bile acid levels, (**B**) conjugated bile acid levels, (**C**) primary bile acid levels, (**D**) secondary bile acid levels, (**E**) endogenous secondary bile acids (excluding UDCA, TUDCA, GUDCA), (**F**) lithocholic acid. Mann-Whitney U test was performed to generate p-values. Only p-values < 0.1 are displayed. All p-values shown are raw/unadjusted. When adjusted, in panels E and F, the day 60 post-HCT FDR-adjusted p-value is 0.02 and 0.16, respectively.

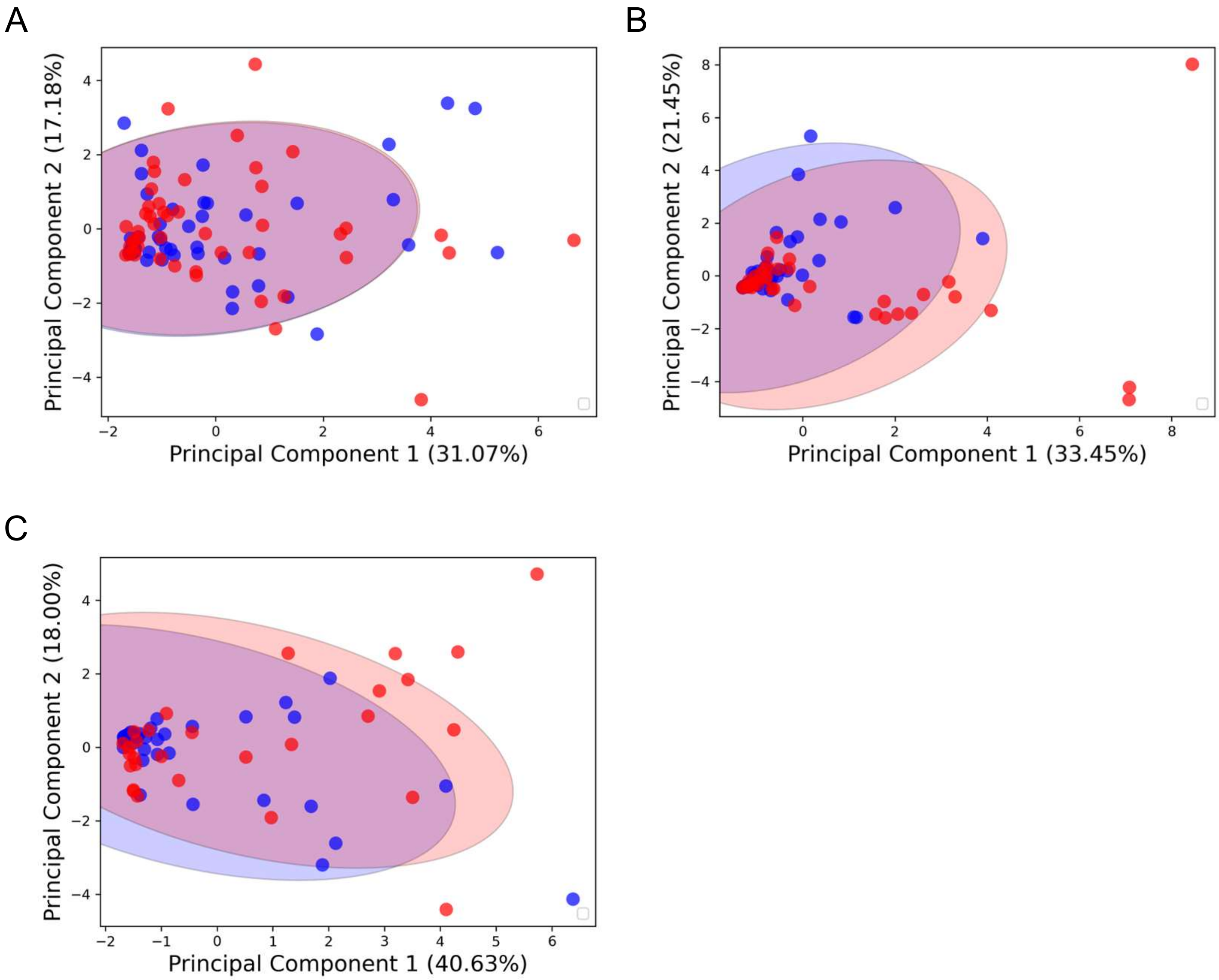

Supplemental Figure 4. Differences in endogenous secondary bile acids. Principal component analysis (PCA) was used to assess differences in the population of 11 endogenous secondary bile acid levels between GvHD cases and controls. (A) Pre-transplant, there were no differences between GvHD cases vs controls. However, PCA revealed statistically significant differences between GvHD cases and controls along Principal Component 2 at (B) 30 days post-transplant (p = 0.009) and along Principal Component 1 at (C) 60 days post-transplant (p = 0.05). Values next to the component on each axis display the amount of variation attributed that component. Cases are in blue, controls in red. All p-values shown are raw/unadjusted. Mann-Whitney U test was performed to generate p-values.

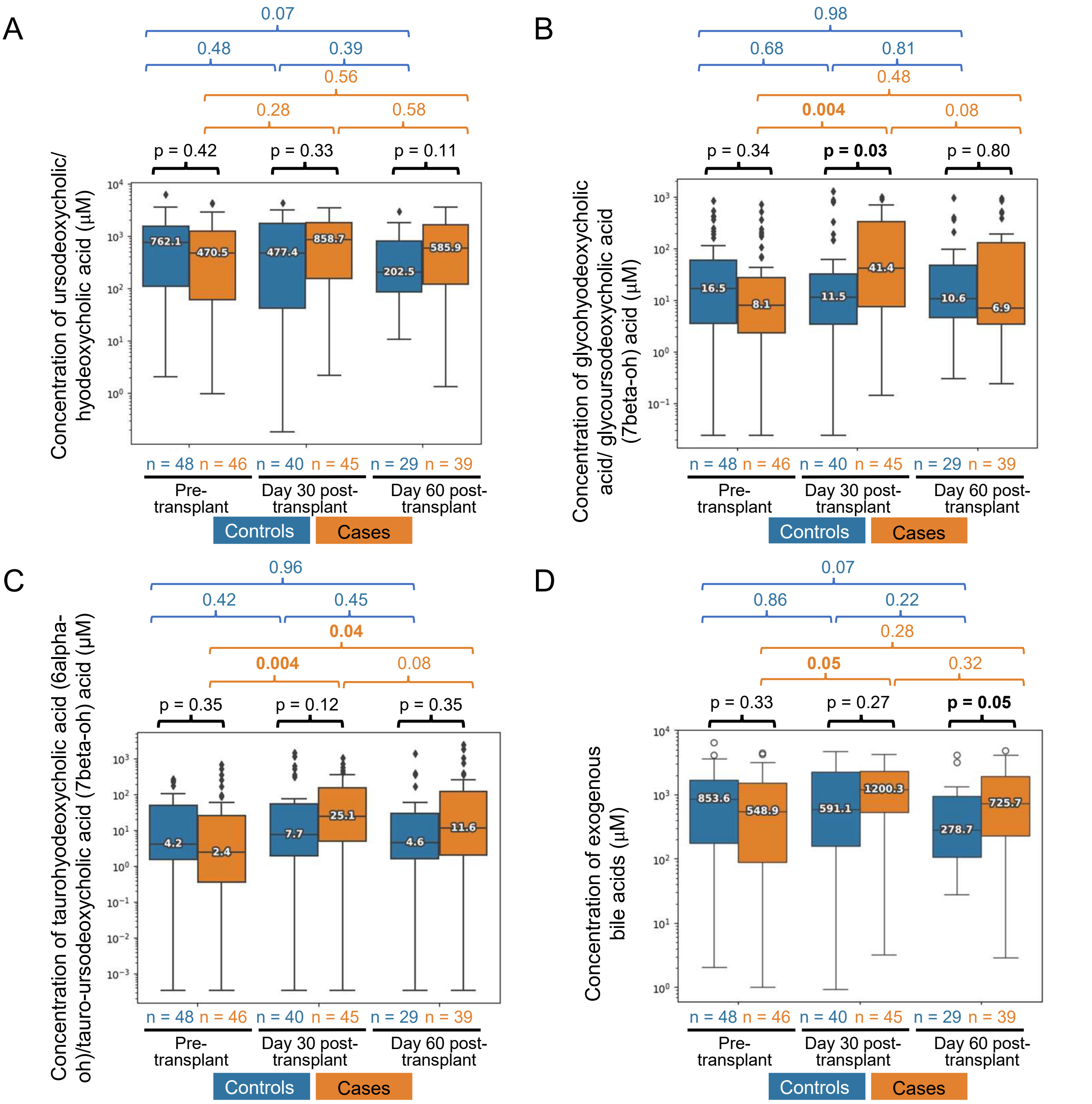

Supplemental Figure 5. Levels of exogenous bile acids. (**A**) Levels of ursodeoxycholic acid and hyodeoxycholic acid, which are indistinguishable from each other by the LC-MS method. (**B**) Levels of glycohyodeoxycholic acid and glycoursodeoxycholic acid (7beta-oh), which are indistinguishable from each other by the LC-MS method. (**C**) Levels of taurohyodeoxycholic acid (6alpha-oh) and tauro-ursodeoxycholic acid (7beta-oh), which are indistinguishable from each other by the LC-MS method. (**D**) Combined levels of ursodeoxycholic acid/hyodeoxycholic acid, glycohyodeoxycholic acid/glycoursodeoxycholic acid (7beta-oh) and taurohyodeoxycholic acid (6alpha-oh)/tauro-ursodeoxycholic acid (7beta-oh). All p-values shown are raw/unadjusted. Mann-Whitney U test was performed to generate p-values.

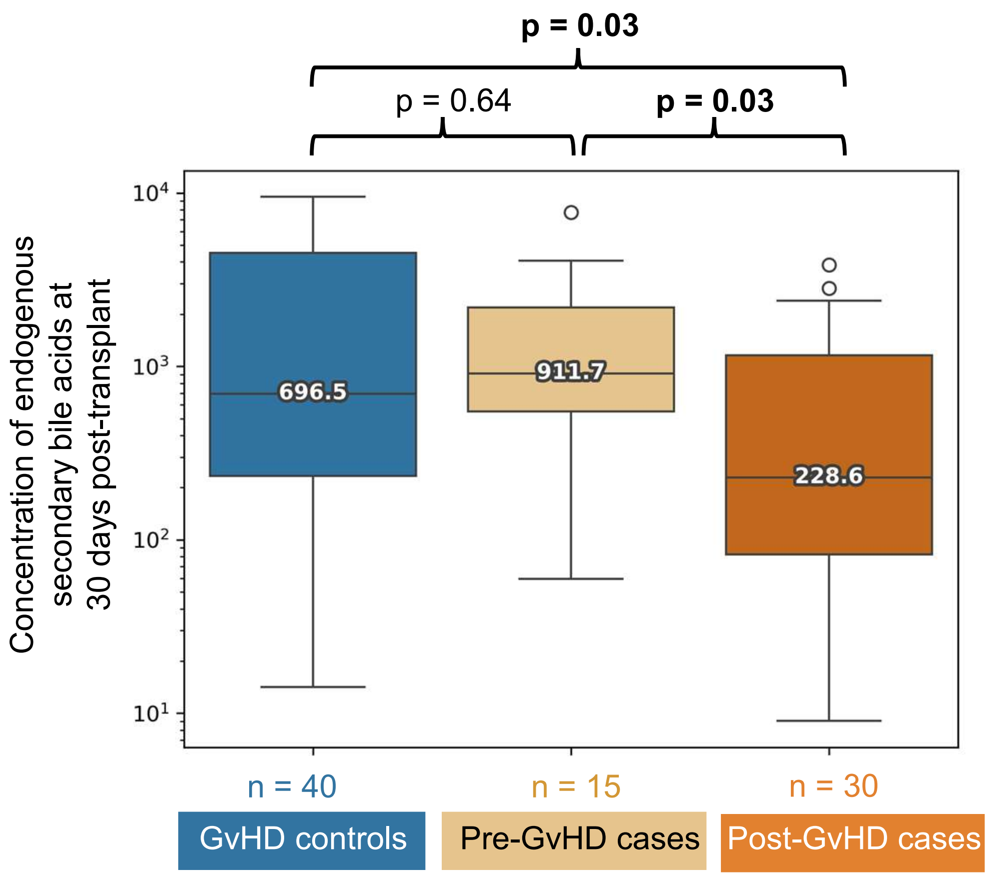

Supplemental Figure 6. Endogenous secondary bile acid levels when adjusted for time to GvHD. At day 30 post-transplant, most of the GvHD case recipients had been diagnosed with GvHD (n = 30), but a minority of the GvHD case recipients had not yet been diagnosed with GvHD (n = 15). Recipient GvHD cases with GvHD already diagnosed at the time of day 30 post-transplant sample had significantly lower levels of endogenous secondary bile acids vs. recipient GvHD cases without GvHD yet and vs. GvHD controls. Recipient GvHD cases without GvHD diagnosed yet had similar levels of endogenous secondary bile acids as GvHD controls. The p-values shown are raw, and when FDR-adjusted, are no longer statistically significant: GvHD controls vs. pre-GvHD cases adjusted p = 0.82, pre-GvHD cases vs. post-GvHD cases adjusted p = 0.22, GvHD controls vs. post-GvHD cases adjusted p = 0.22. Mann-Whitney U test was performed to generate p-values.

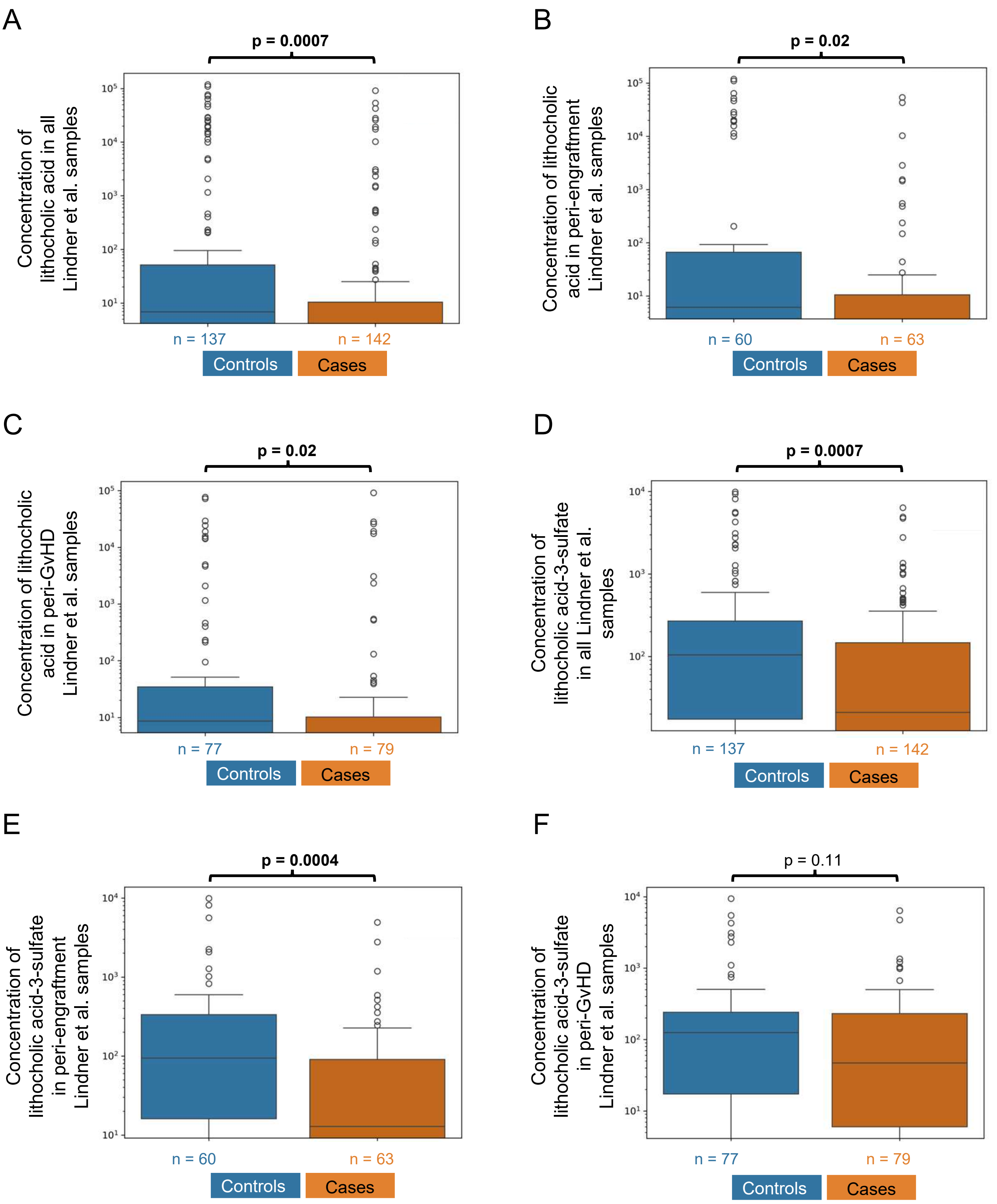

Supplemental Figure 7. Findings from a similar study are concordant with our observations. A publicly available dataset from Lindner et al. (2024) in human HCT-recipients with and without GvHD validate our study's findings that lithocholic acid and similarly structured bile acids are lower in GvHD vs. no GvHD. Lithocholic acid concentrations from Lindner et al. (2024) samples (**A**) overall, (**B**) peri-engraftment, and (**C**) peri-GvHD. Lithocholic acid-3-sulfate concentrations from Lindner et al. (2024) samples (**D**) overall, (**E**) peri-engraftment, and (**F**) peri-GvHD. All p-values shown are raw/unadjusted. Mann-Whitney U test was performed to generate p-values.

A

B

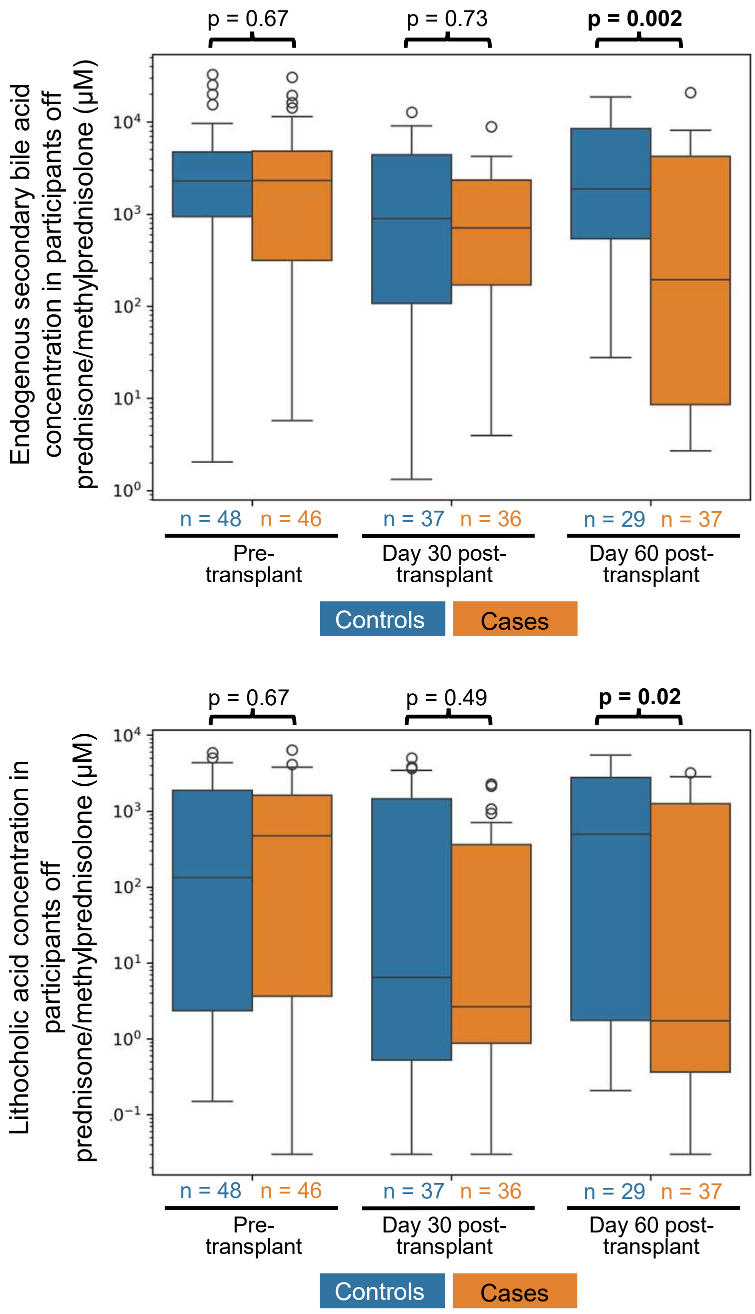

Supplemental Figure 8. Prednisone/methylprednisolone therapy does not bias bile acid levels. To investigate if prednisone/methylprednisolone treatment biases the observed bile acid levels in stool, we removed the 14 samples collected from patients that were actively on this steroid therapy. We defined this as having received prednisone or methylprednisolone in the seven or fewer days prior to sample date. Key findings were replicated without the effect of this steroid therapy. (A) Endogenous secondary bile acid and (B) lithocholic acid, an endogenous secondary bile acid, concentrations were statistically significantly reduced in GvHD cases vs. controls at day 60 post-HCT. Statistical significance of these differences was not impacted when considering steroid use. This supports that prednisone/methylprednisolone steroid therapy does not bias bile acid levels. All p-values shown are raw/unadjusted. Mann-Whitney U test was performed to generate p-values.

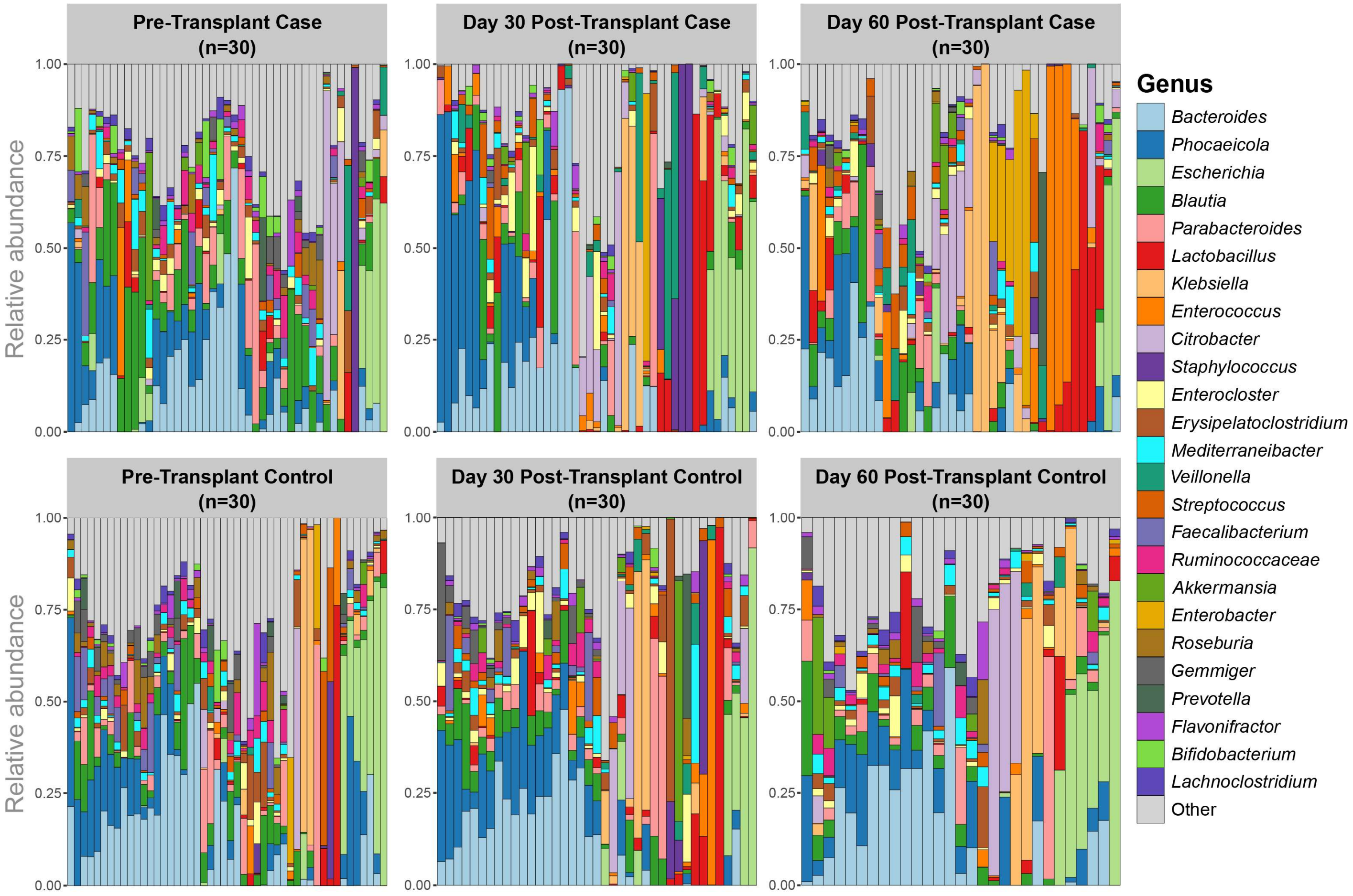

Supplemental Figure 9. Relative abundance of top 20 bacterial genera calculated from 16S rRNA gene sequencing data. There are global similarities between GvHD case and control stool samples. We observed no specific taxa at the family, genus or species level that was statistically significantly different by GvHD status when using the Mann Whitney U test and when adjusting for multiple comparisons.

Supplemental Table 3. Concentrations of endogenous secondary bile acids. Median concentrations (μM), raw p-values and adjusted p-values from endogenous secondary bile acids and key comparisons by GvHD status and timepoints throughout the HCT process. Mann-Whitney U test was performed to generate p-values.

	Pre-Tx				Day 30				Day 60			
	median (μM)			adj.	median (μM)			adj.	median (μM)			adj.
	control	case	p-value	p-value	control	case	p-value	p-value	control	case	p-value	p-value
12KLCA814 (3alpha-hydroxy-12 ketolithocholic acid)	182.7	139.8	0.483	0.693	0.2	0.2	0.124	0.37	54.3	0.2	0.029	0.157
7KLCAolone (3alpha-hydroxy-7-ketolithocholic acid)	419.1	137.1	0.213	0.503	175.9	187.7	0.478	0.693	169.4	49.7	0.035	0.167
5beta-cholanic acid-3beta, 12alpha-diol	25.2	11.7	0.107	0.354	8.3	7.2	0.824	0.938	19.5	4.4	0.011	0.157
allolithocholic acid	0	0.6	0.46	0.693	0	0	0.021	0.157	0.4	0	0.101	0.354
deoxycholic acid	142.9	75.3	0.96	0.997	0.1	0.1	0.301	0.552	15.7	0.1	0.146	0.37
glycodeoxycholic acid	0.4	0.5	0.997	0.997	0	0	0.973	0.997	0.5	0	0.238	0.507
glycolithocholic acid	3	3.9	0.342	0.595	2.7	1.9	0.027	0.157	1.7	1.9	0.76	0.896
isolithocholic acid	8.9	35.4	0.363	0.6	3	1.6	0.249	0.507	237.4	4.8	0.14	0.37
lithocholic acid	134.2	475.4	0.669	0.849	5.2	1.6	0.261	0.507	497.8	1.9	0.02	0.157
taurodeoxycholic acid	0.3	0.1	0.604	0.797	0	0	0.547	0.753	0.1	0.5	0.867	0.954
taurolithocholic acid	0.4	0.4	0.743	0.896	0.6	0.2	0.013	0.157	0.4	0.2	0.054	0.222
Total endogenous secondary bile acids	1891.1	1521.3	0.568	0.789	696.5	622.9	0.158	0.439	1848	429.6	0.004	0.019

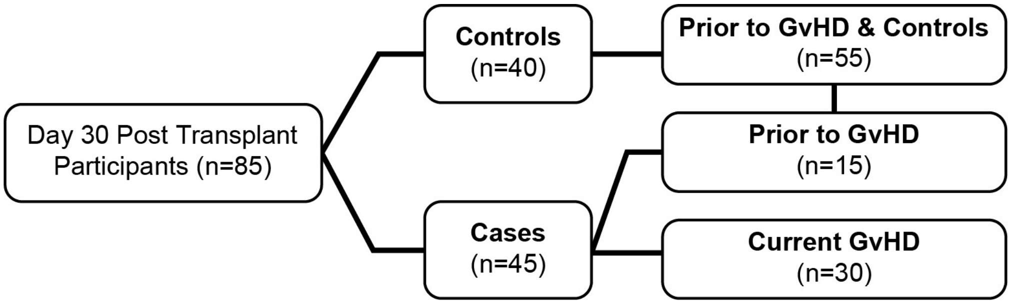

	Median (µM)			Comparisons							
				**Prior to GvHD** vs. **Current GvHD**		**Prior to GvHD** vs. **Controls**		**Current GvHD** vs. **Controls**		**Current GvHD** vs. **Prior to GvHD & Controls**	
	**Prior to GvHD** (n=15)	**Current GvHD** (n=30)	**Prior to GvHD & Controls** (n=55)	p-value	adj. p-value	p-value	adj. p-value	p-value	adj. p-value	p-value	adj. p-value
12KLCA814 (3alpha-hydroxy-12 ketolithocholic acid)	0.2	0.2	0.2	0.509	0.784	0.549	0.784	0.093	0.388	0.121	0.403
7KLCAolone (3alpha-hydroxy-7-ketolithocholic acid)	538.5	108.3	230.4	0.148	0.435	0.101	0.388	0.948	0.995	0.543	0.784
5beta-cholanic acid-3beta, 12alpha-diol	10.6	3.6	9.4	0.112	0.4	0.209	0.523	0.627	0.824	0.323	0.66
allolithocholic acid	0	0	0	0.068	0.378	0.462	0.745	0.008	0.113	0.009	0.113
deoxycholic acid	0.1	0.1	0.1	0.963	0.995	0.533	0.784	0.33	0.66	0.433	0.745
glycodeoxycholic acid	0	0	0	0.957	0.995	0.958	0.995	0.995	0.995	0.926	0.995
glycolithocholic acid	1.6	2.1	2.3	0.462	0.745	0.048	0.3	0.083	0.388	0.29	0.631
isolithocholic acid	0.1	1.6	2	0.721	0.901	0.241	0.574	0.418	0.745	0.623	0.824
lithocholic acid	1.9	1.6	3.9	0.263	0.598	0.813	0.945	0.174	0.458	0.137	0.429
taurodeoxycholic acid	0	0	0	0.746	0.91	0.41	0.745	0.782	0.931	0.992	0.995
taurolithocholic acid	0.4	0.1	0.6	0.095	0.388	0.356	0.685	0.006	0.113	0.006	0.113
Total endogenous secondary bile acids	911.7	228.6	713.3	0.031	0.221	0.643	0.824	0.028	0.221	0.011	0.113

Supplemental Table 4. Concentrations and comparisons of endogenous secondary bile acids on day 30 post-HCT. Median concentrations (µM), raw p-values and adjusted p-values from endogenous secondary bile acids and key comparisons by GvHD status on day 30 post-transplant. Sample sizes (n) are displayed in parentheses in the column headers. At this day 30 post-HCT timepoint, there were 40 GvHD control participants and 45 GvHD case participants. Within the 45 cases, there were 15 GvHD case participants without GvHD diagnosed at the time of sample (“Prior to GvHD”) and 30 GvHD control participants with GvHD diagnosed at the time of sample (“Current GvHD”). There were 55 total participants without GvHD at the time of sample (15 cases Prior to GvHD + 40 Controls). Mann-Whitney U test was performed to generate p-values.

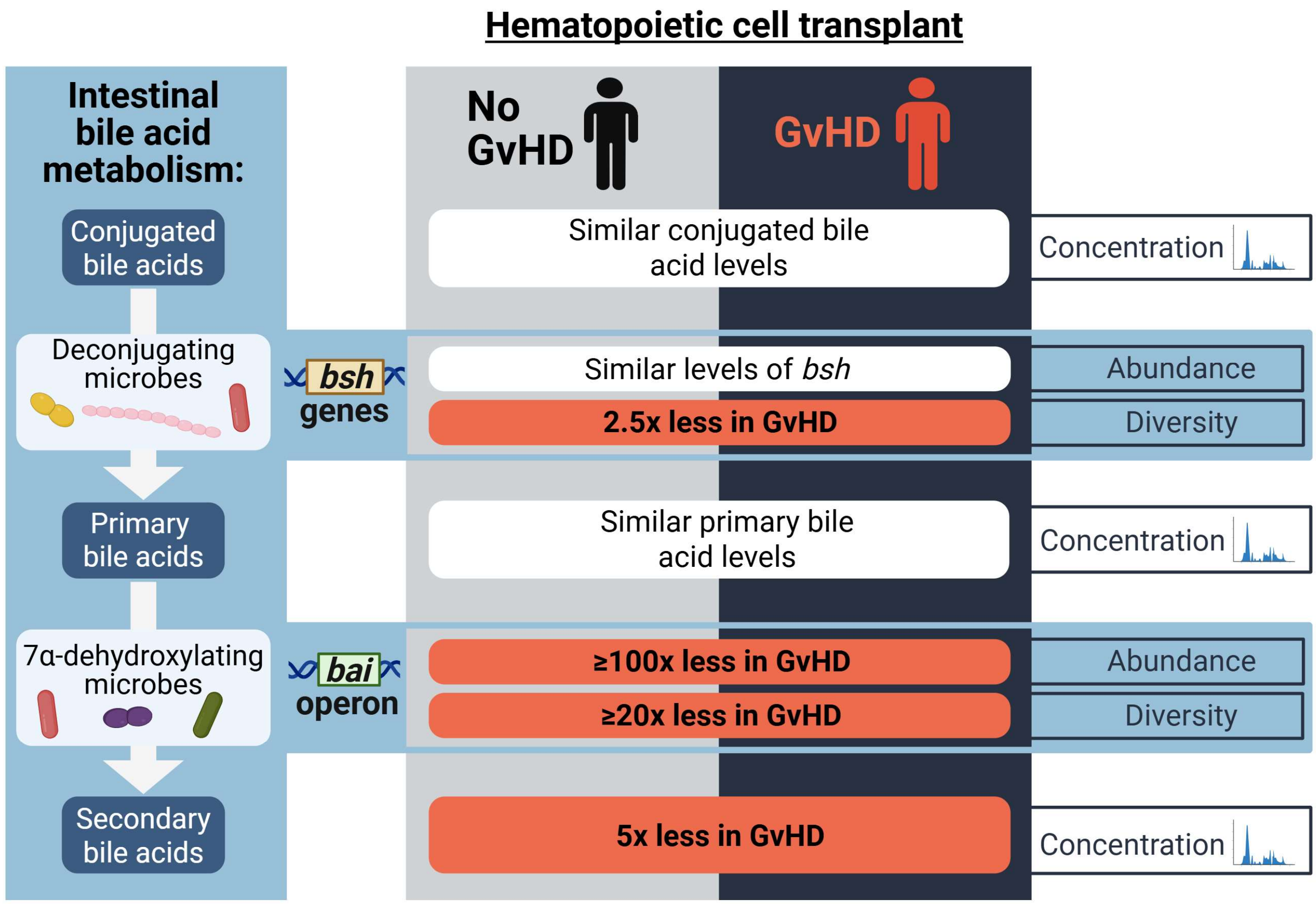

Supplemental Figure 10. Acute gastrointestinal GvHD is associated with reductions of secondary bile acids, *bsh* genes, and *bai* genes after allo-HCT. This visual abstract summarizes the findings of our study. Created in BioRender. Proll, S. (2026) https://BioRender.com/5z04gp7.
